# Capacitively Coupled Plasma Discharge of Ionic Liquid Solutions to Synthesize Carbon Dots as Fluorescent Sensors

**DOI:** 10.3390/nano8060372

**Published:** 2018-05-26

**Authors:** Ching-Bin Ke, Te-Ling Lu, Jian-Lian Chen

**Affiliations:** 1 Department of Beauty and Health Care, Min-Hwei Junior College of Health Care Management, No. 1116, Sec. 2, Zhongshan E. Rd., Tainan 73658, Taiwan; cbke@mail.mhchcm.edu.tw; 2 School of Pharmacy, China Medical University, No. 91 Hsueh-Shih Road, Taichung 40402, Taiwan; lutl@mail.cmu.edu.tw

**Keywords:** capacitively coupled plasma, carbon dots, ionic liquid, mercury ion, quercetin, upconversion

## Abstract

Oxygen and nitrogen capacitively coupled plasma (CCP) was used to irradiate mixtures of aliphatic acids in high boiling point solvents to synthesize fluorescent carbon dots (C-dots). With a high fluorescence intensity, the C-dots obtained from the O_2_/CCP radiation of a 1-ethyl-3-methylimidazolium dicyanamide ionic liquid solution of citric acid were characterized with an average diameter of 8.6 nm (σ = 1.1 nm), nitrogen and oxygen bonding functionalities, excitation-independent emissions, and upconversion fluorescence. Through dialysis of the CCP-treated C-dots, two emissive surface states corresponding to their respective functionalities and emissions were identified. The fluorescence spectrum of the CCP-treated C-dots was different from that of the microwave irradiation and possessed higher intensity than that of hydrothermal pyrolysis. By evaluation of the fluorescence quenching effect on flavonoids and metal ions, the CCP-treated C-dots showed a high selectivity for quercetin and sensitivity to Hg^2+^. Based on the Perrin model, a calibration curve (*R*^2^ = 0.9992) was established for quercetin ranging from 2.4 μM to 119 μM with an LOD (limit of detection) = 0.5 μM. The quercetin in the ethanol extract of the sun-dried peel of *Citrus reticulata* cv. Chachiensis was determined by a standard addition method to be 4.20 ± 0.15 mg/g with a matrix effect of 8.16%.

## 1. Introduction

Carbon dots (C-dots) have readily acquired predominance over commercial dyes and conventional semiconductor quantum dots in a wide variety of analytical and biomedical applications, including (bio)chemical sensing, photocatalysis, electrochemiluminescence, photoelectrochemical sensing, bioimaging, and drug delivery, because of their tunable photoluminescence, fine resistance to photobleaching, excellent water solubility, good biocompatibility, and ease of synthesis [1,2,3,4]. Based on the chosen carbonaceous precursors, the approaches to synthesize C-dots are divided into top-down and bottom-up routes [3,4,5,6]. The former involves cleaving or breaking down relatively macroscopic carbonaceous materials, such as carbon nanotubes, graphite columns, and graphene powder, via acidic and electrochemical oxidation, arc-discharge, and laser ablation. The latter is realized through carbonization of small organic molecules via solvothermal and hydrothermal pyrolysis, microwave and ultrasonic irradiation, and plasma treatment. The low requirement for carbon molecules in the bottom-up route is advantageous for obtaining C-dots with desirable morphological, functional, and spectral properties.

Due to equipment availability, plasma treatment is less reported than other approaches, especially compared to the mainly utilized microwave and hydrothermal syntheses. However, the ability to control the nanoscale localization of energy and matter delivered from bulk plasmas to form nano-solids is capable of unique self-organized processes [7]. In 2010, C-dots were first generated by the reaction of benzene with atmosphere helium cavitation gas in a submerged-arc plasma reactor [8]. Another plasma–liquid system used argon atmospheric-pressure microplasma as gaseous electrodes to purge an aqueous solution containing citric acid and ethylenediamine to produce C-dots [9]. Without the need for a well-designed reactor, the plasma-gas system can use gaseous and solid organic precursors. Methane, hydrogen, and nitrogen were used as the reactive gases in a plasma-enhanced hot filament chemical vapor deposition of carbon nanofilms and nanodots [10]. Ethylene gas was introduced continuously along with the thermal argon plasma jet at sound velocity to produce graphene quantum dots [11,12]. Also, solid egg white, yolk, acrylamide, and ashes of plant leaves were irradiated by air atmospheric-pressure dielectric barrier discharge plasmas to generate C-dots [13,14,15]. No liquid precursors have been irradiated in a plasma-gas system to obtain C-dots. 

In this study, aliphatic acids dispersed in viscous media, ionic liquids and high b.p. solvents, were irradiated with capacitively coupled plasma (CCP) between regular plate electrodes in a low-pressure chamber, in presence of oxygen; to obtain C-dots. Due to its high fluorescence intensity, the C-dots synthesized via O_2_/CCP treatment of 1-ethyl-3-methylimidazolium dicyanamide solution of citric acid were further characterized to determine the physical dimensions, crystallinity, functionality, and spectral properties of the C-dots. A possible fluorescence mechanism was proposed. After comparison with hydrothermal and microwave treatments, the O_2_/CCP-treated C-dots were applied to detect metal ions and flavonoids. 

## 2. Materials and Methods

### 2.1. Materials and Chemicals

Citric acid was purchased from Acros (Thermo Fisher Scientific, Geel, Belgium). Malic acid, ethylene glycol, 1,4-butanediol, and poly(ethylene imide) (PEI, branched, Mn = 600, Mw = 800) were from Aldrich (Milwaukee, WI, USA). Poly(ethylene glycol) (PEG, Mn = 1000) was purchased from Alfa Aesar (Ward Hill, MA, USA). Adipic acid, succinic acid, and glycerol were from Showa (Tokyo, Japan). Ionic liquids were purchased from Aldrich (1-ethyl-3-methylimidazolium dicyanamide, [EMIM]N(CN)_2_; 1-ethyl-3-methylimidazolium tetrachloroaluminate, [EMIM]AlCl_4_; 1-methyl-3-octylimidazolium chloride, [MOIM]Cl); and trihexyltetradecylphosphonium dicyanamide, [P6,6,6,14]N(CN)_2_) and Alfa Aesar (1-butyl-3-methylimidazolium tetrafluoroborate, [BMIM]BF_4_; 1-butyl-3-methylimidazolium chloride, [BMIM]Cl; and 1-butyl-3-methylimidazolium bromide, [BMIM]Br). Nine flavonoids (5-methoxyflavone (Met), hesperidin (Hpd), naringin (Nag), catechin (Cat), epicatechin (Epi), hesperetin (Hpt), daidzein (Dai), naringenin (Nar), and quercetin (Que)) were from Aldrich. All metal and phosphate salts were of analytical grade and were obtained from Aldrich and Acros. Purified water (18 MΩ∙cm) from a Milli-Q water purification system (Millipore, Bedford, MA, USA) was used to prepare the standard salt and buffer solutions. All standard solutions were protected from light and kept at 4 °C in a refrigerator.

### 2.2. Synthesis and Characterization of C-Dots

For the capacitively coupled plasma (CCP) treatment, a crucible containing a mixture of an aliphatic acid (100 mg) and a viscous solvent (500 μL) was placed on a flat aluminum tray adapted for a cylindrical, stainless-steel, low pressure chamber (diameter of 10 cm and depth of 27.8 cm), which was evacuated by a rotary vane pump (3.0 m^3^∙h^−1^) in a modular plasma system (Femto SRS, Diener electronic GmbH + Co. KG, Ebhausen, Germany). The system was initiated by an RF generator (13.56 MHz, 0~100 W) to discharge the inlet gas (O_2_ or N_2_) between an aluminum planar electrode and the flat tray under a working pressure of 0.285 torr. The gas flow was manually adjusted by a needle valve (0~50 sccm), and the chamber pressure was measured by a Pirani sensor (10^−2^~10 mbar). The small-footprint, table-top system was semi-automatically processed through evacuation pumping, gas inlet, plasma ignition, and ventilation. For comparison, the crucible was placed in a domestic microwave oven (0~1150 Watt, 2450 MHz) or in a Teflon-lined vessel, sealed in a stainless-steel autoclave (50 mL) and calcined at a constant temperature (200 °C) for a specified period of time.

After treatment, the crucible was rinsed with 1.0 mL of H_2_O to collect the synthesized C-dot products. The 1.0 mL C-dot solutions were diluted at different ratios with water for characterization or with phosphate buffers (50 mM, pH 7.0) to build a calibration curve. The diluted aqueous solutions were characterized by a ultraviolet-visible (UV-Vis) spectrometer (Lambda 35, Perkin Elmer, Cambridge, MA, USA), spectrofluorometer (LS55, Perkin Elmer, Cambridge, MA, USA), Fourier transform infrared (FTIR) spectrometer (Prestige-21, Shimadzu, Japan) equipped with a single reflection horizontal ATR accessory (MIRacle, PIKE Technologies, Fitchburg, WI, USA), high-resolution transmission electron microscopy (JEM-2100, JEOL, Tokyo, Japan) operated at an accelerating voltage of 200 kV, and laboratory-built electrophoresis apparatus consisting of a ±30 kV high-voltage power supply (TriSep TM-2100, Unimicro Technologies, Pleasanton, CA, USA) and a UV-Vis detector (LCD 2083.2 CE, ECOM, Prague, Czech). Some product solutions were dried to evaporate the water at 70 °C in a vacuum oven for 24 h to prepare solid samples for high-resolution X-ray diffraction (D8 Discover, Bruker, MA, USA) and X-ray photoelectron spectroscopy (ULVAC-PHI PHI 5000 VersaProbe, Physical Electronics, Eden Prairie, MN, USA). Some product solutions (1.0 mL) were dialyzed against ultra-pure water through a molecular weight cutoff membrane (500–1000 Dalton, Float-A-Lyzer G2, 1 mL capacity, Spectrum Laboratories Inc., Savannah, GA, USA) to study the effect of the dialysis on the separation of the C-dot products.

### 2.3. Fluorescence Measurement of Samples with the CCP-Treated C-Dots

The CCP treatment of the mixture of citric acid (100 mg) and the ionic liquid (500 μL) [EMIM]N(CN)_2_ was performed with 10 sccm O_2_ inlet, 90 Watt RF power, and a 30 min duration. The as-prepared 1.0 mL C-dot solution possessed high fluorescence intensity and was capable of sensing samples. A small volume (0.2 μL) of the C-dot solution was added to each sample containing metal ions (33.3 μM) or flavonoids (8.33 μg/mL) in 3.0 mL of phosphate buffer (50 mM, pH 7.0) to measure the fluorescence intensities (*I*) at 430 nm (excitation at 330 nm) and 480 nm (excitation at 390 nm). These *I* values were compared with those (*I*_0_) observed in the blank samples to evaluate the quenching effect (*I*/*I*_0_) on the samples and to further establish the calibration curves for quercetin, a flavonoid.

A flavonoid-rich traditional Chinese medicine (TCM), called “Guang-Chen-Pi” in Chinese, was purchased from a TCM store in Taichung, Taiwan, and the amount of quercetin in the medicine was determined. After cleaning with deionized water, 5.0 g of dried Guang-Chen-Pi was triturated and refluxed in 50 mL ethanol for two hours. After filtering through a glass microfiber disc (GF/A, Whatman, England), 25 mL of the filtrate was concentrated to dryness on a rotary evaporator. The remaining residue was dissolved with 7.2 mL ethanol and became the sample solution. Five 3.0 mL vials were each spiked with 6.0 μL of the sample solution, and four of the five vials were further spiked with 1.0, 2.0, 3.0, and 4.0 μL of the quercetin standard solution (7.24 × 10^−3^ M). The quercetin in the ethanol extract of the Guang-Chen-Pi was calculated from the standard addition curve established by the *I* values of the five vials.

## 3. Results and Discussion

### 3.1. Choice of Short Aliphatic Acids and Solvents

Four short aliphatic acids, including citric acid (CA), succinic acid, adipic acid, and malic acid (MA), were separately dispersed in high b.p. solvents, including glycerol, PEG 1000, PEI 600, and seven ionic liquids, which are listed in Section 2.1. Then, the mixtures were treated with the oxygen-gas (10 sccm), RF-discharge (90 Watt) plasma produced between the capacitive-coupling, parallel-plate electrodes. If lower b.p. solvents, such as ethylene glycol (b.p. 197 °C) and 1,4-butanediol (b.p. 235 °C), were substituted for the high b.p. solvents, a large amount of vaporized solvent was rapidly produced just after the plasma initiation, and the vacuum pumping and RF power were quickly stopped. If only the acids were treated by the capacitively coupled plasma (CCP) without the addition of solvents, the white powder products looked like the native, untreated acids and showed no fluorescence. After dispersion in the high b.p. solvents and a 30 min treatment with O_2_/CCP, dark brown products were obtained, re-dispersed in water, and measured by UV-Vis and fluorescence spectroscopies. The observed spectra are shown in Table S1 in the Electronic supplementary information (ESI). Except for glycerol and PEG 1000, for which the CCP products did not show any fluorescence, most of the solvents had excitation-dependent emissions. Glycerol and PEG do not contain any nitrogen atoms in their chemical structures, but the other solvents do. The nitrogen involvement in O_2_/CCP carbonization of the acids is crucial for obtaining fluorescent C-dots. Although some fluorescent C-dots were obtained from non-nitrogen-containing materials by solvothermal [16], pyrolysis [17], hydrothermal [18], and microwave heating [19], nitrogen gas in air can enter the heating apparatuses and encounter the reactants to introduce nitrogen atoms into the C-dot structures. Even heating glycerol or PEG solvents alone in a domestic microwave oven resulted in fluorescent C-dots [20,21]. In this study, the nitrogen in air could not enter the CCP vacuum chamber. The only nitrogen source was the high b.p. solvents. Currently, most fluorescent C-dots are synthesized from nitrogen-containing carbon sources, solvents, or additives. In these cases, nitrogen-containing solvents act both as a dispersant and a passivant.

**Figure 1:**
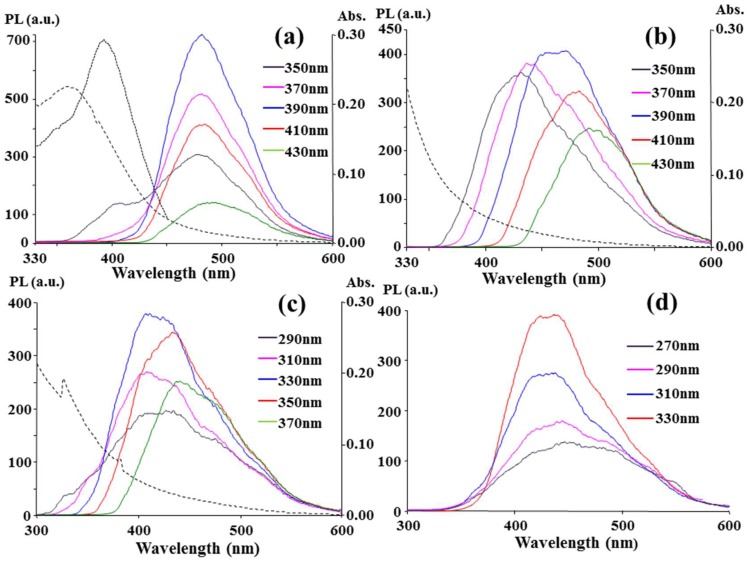
Fluorescence spectra of (**a**,**d**) CA + [EMIM]N(CN)_2_; (**b**) [EMIM]N(CN)_2_; and (**c**) CA + glycerol treated by O_2_/CCP (**a**,**b**,**d**) and N_2_/CCP (**c**). The mixtures of CA (100 mg) and solvents (500 μL) were treated by CCP (10 sccm of gas flow, RF power 90 W, 30 min). After the addition of 1.0 mL of H_2_O to the treated products, different volumes of the product solutions (0.2 μL for (**a**,**d**) and 20 μL for (**b**,**c**) were separately transferred to cuvettes filled with 3.0 mL of H_2_O for spectrometric measurements. (---) and (∙∙∙∙) curves denote UV-Vis absorption and PL (emission at 480 nm) spectra, respectively.

As shown in Table S1, the highest photoluminescence (PL) intensity (*I*_max_) observed at the corresponding emission (λ_em,max_) and excitation (λ_ex,max_) wavelengths varied with the acid and nitrogen-containing solvent used. Some of the solvents, including PEI 600, [BMIM]AlCl_4_, [BMIM]BF_4_, and [MOIM]Cl, possessed similar λ_em,max_ and λ_ex,max_ values for all the acids, but other solvents did not. Most of the CCP products possessed an obvious dependence of λ_em_ on λ_ex_, but a few of them did not. Interestingly, the C-dots obtained from the O_2_/CCP treatment of CA and MA in [EMIM]N(CN)_2_ possessed the first and second highest photoluminescence (PL) intensities among the CCP products in Table S1, but their dependence of λ_em_ on λ_ex_ was obscure. As shown in Figure 1a and Table S1, the emission redshifts were only 11 nm and 26 nm as an increase of 80 nm in λ_ex_ was applied to the CA-based C-dots (350 nm to 430 nm) and MA-based C-dots (300 nm to 380 nm), respectively. In contrast, as shown in Figure 1(b), an obvious 58 nm redshift emission (λ_ex_ = 350~430 nm) was observed for the O_2_/CCP of [EMIM]N(CN)_2_ alone. Moreover, the λ_em,max_ position and the smaller PL intensity in Figure 1b were different from those of the CA- and MA-based C-dots. There should be a synergistic effect of [EMIM]N(CN)_2_ and the acids, CA and MA, on the formation of fluorescent C-dots in the O_2_/CCP carbonization. Further discussion about the independence of λ_ex_ for the CA-based C-dots is addressed in Section 3.2.

Instead of O_2_/CCP, the N_2_/CCP treatment of CA in glycerol under the same CCP conditions converted non-fluorescent C-dots to fluorescent ones, as shown in Figure 1c, and they present a λ_em,max_ of 400 nm with a λ_ex,max_ of 330 nm and a 19 nm redshift emission with an excitation from 290 nm to 370 nm. The definite difference in the fluorescence and UV-Vis spectra between Figure 1a,c implies the different routes of C-dot formation. Although the N_2_/CCP treatment aided the use of non-nitrogen-containing glycerol, its PL intensity was much lower than that of the product from the O_2_/CCP treatment of CA in [EMIM]N(CN)_2_ at the same dilution factor. Because a fluorophore with a high PL intensity is advantageous for analytical uses, CA dispersed in [EMIM]N(CN)_2_ was selected to be the carbon source for the O_2_/CCP carbonization in the following discussion.

### 3.2. Characterization of CCP-Treated C-Dots

**Figure 2:**
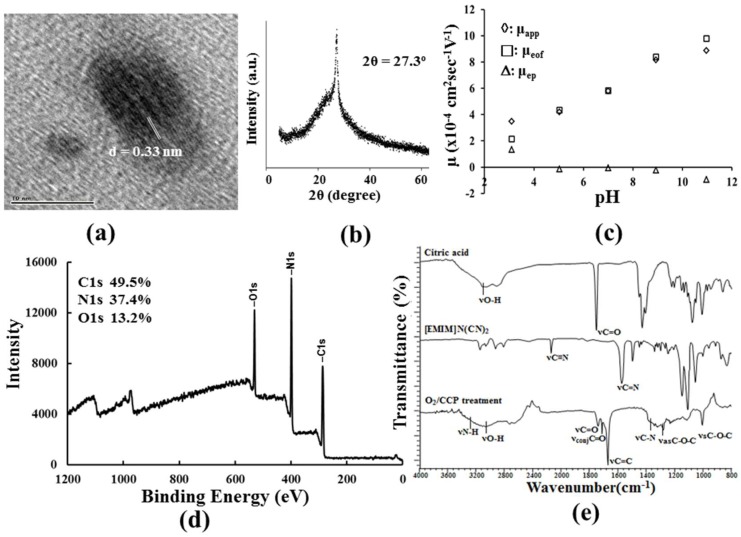
Characterization of the C-dots prepared in the conditions of Figure 1a. (**a**) HRTEM image; (**b**) XRD spectrum; (**c**) the plot of mobility with the pH of the running buffers; (**d**) XPS spectrum, and (**e**) FTIR spectrum.

The high-resolution TEM (HRTEM) image of the C-dots obtained in the conditions of Figure 1a is shown in Figure S1, and the average size of the 55 particles analyzed by the ImageJ freeware was 8.6 nm (σ = 1.1 nm). Most of the C-dots did not have any clear lattice fringes in the HRTEM image, but a few indicated spaces between the graphene layers, as shown in Figure 2a and Figure S1. This was further supported by the X-ray diffraction (XRD) pattern in Figure 2b, which displayed a broad diffraction peak due to the amorphous nature of the sample and a distinct peak centered at 2θ = 27.3° for the (002) facet of graphite. The prepared C-dots would be classified as carbon quantum dots rather than carbon nanodots according to the classification in a recent paper [22]. Capillary zone electrophoresis was used to analyze the changes in the surface charge of the C-dots via the electrophoretic mobility (μ_ep_) at the pH of the running buffers. As shown in Figure 2c, the μ_ep_ values, which were determined by subtracting the electroosmotic mobilities (μ_eof_) from the apparent mobilities (μ_app_) and inferred net charge on the C-dot surface, were positive below pH 5.0 and negative above pH 8.9. The dissociation of carboxylic acid and protonated amine or 1.3-diketones would occur on the C-dot surface as their pKa values are near to 5.0 and 8.9, respectively. The full scan of the XPS spectrum presents the main peaks of C 1s, N 1s, and O 1s in Figure 2d. The carbon content (49.5%) in the O_2_/CCP product was reasonable and in the range between the reactants CA (C: 37.5%) and [EMIM]N(CN)_2_ (C: 58.9%). However, a large decrease in the oxygen percentage from 58.3% in CA to 13.2% in the product was distinct from the slight increase in the nitrogen percentage from 34.4% in [EMIM]N(CN)_2_ to 37.4% in the product. This indicates that the oxygen atoms in CA were more easily subtracted by the oxygen plasma than the nitrogen atoms bound in the imidazole ring in [EMIM]N(CN)_2_. The deconvolution of the main peaks is shown in Figure S2. In detail, four peaks related to the C–C (284.3 eV), C–N (284.9 eV), C–O (285.9 eV), and C=O/C=N (287.6 eV) functional groups were deconvoluted from the C 1s spectrum. The N 1s spectrum contained three nitrogen bonding groups, including C–N–C (399.5 eV), N–(C)_3_ (400.2 eV), and N–H (401.9 eV). The O 1s spectrum contained two characteristic peaks corresponding to the C=O (530.7 ev) and C–OH/C–O–C (532.3 eV) groups. Although the peaks of C=N and N–(C)_3_ may support the existence of the imidazole group, the peaks for C–N, C–N–C, and N–H imply that parts of the imidazole rings were pyrolyzed. Further evidence of the decomposition of imidazole rings was given by the FTIR spectra in Figure 2e, which show that the characteristic absorption bands of the aromatic CN heterocycles observed at 1465 to 1600 cm^−1^ in [EMIM]N(CN)_2_ disappeared after the O_2_/CCP treatment. Even the dicyanamide anion decomposed, and its C≡N group absorption at 2145 cm^−1^ also vanished after the plasma action. The other typical peaks of the plasma product were recognized as specific chemical bonding in Figure 2e.

The PL, PLE (PL excitation), and UV-Vis absorption spectra of the as-prepared C-dots are shown in Figure 1a, and an excitation-independent PL and an absorption peak at 360 nm, which was different from λ_ex,max_ = 390 nm, were observed. The emission at approximately 480 nm is independent of the excited light beam from 350 nm to 430 nm. At λ_ex,max_ = 390 nm, the quantum yield of the C-dots was 12.4% based on a calibration against the reference quinine sulfate in 0.5 M H_2_SO_4_, as shown in Figure S3. The same phenomenon occurred in Figure 1d, and the excited light beam from 270 to 330 nm produced another independent emission at approximately 430 nm for the C-dots. At λ_ex,max_ = 330 nm, the quantum yield was calculated to be 7.2% with reference to 2-aminopyridine in 0.5 M H_2_SO_4_, as shown in Figure S3. The two λ_ex_-independent emissions at 430 nm and 480 nm indicate two emissive states for each uniform energy distribution on the C-dots. Neither of the two emissions could be categorized as an intrinsic emission (π*→π) from the carbon core because the quantum confinement effect determines that 8.6 nm in diameter C-dots would have a longer-wavelength emission than near infrared but not visible emissions [23]. The molecule states, which are determined solely by the fluorescent molecules connected on the surface or interior of the C-dots, may be emissive states because λ_ex_ independence is a characteristic of a molecule state [24,25,26,27]. However, the possibility of forming molecule states on the C-dots was excluded because no reasonable route to form a fluorophore molecule via the reaction of CA and [EMIM]N(CN)_2_ could be inferred, whereas fluorophores were reasonably formed by the reaction of CA and a primary amine, such as ethanolamine and ethylene diamine [24,27]. Therefore, the two emissions should come from the surface states, which consisted of hybridization of the carbon backbone and the connected chemical groups because the π* and molecule states were excluded as the emissive states [28].

**Figure 3:**
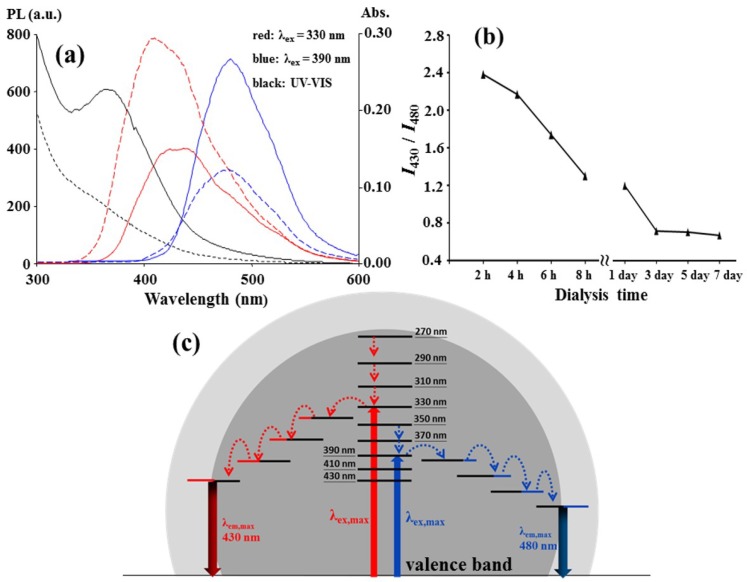
(**a**) Fluorescence and UV-Vis absorption spectra of the C-dots prepared in the conditions of Figure 1a before (solid lines) and after (dashed lines) dialysis in H_2_O by a 500–1000 Da cut-off membrane for two hours; (**b**) The plot of the PL intensity ratios of 430 nm PL intensity to 480 nm PL intensity with the dialysis duration; (**c**) The proposed PL mechanism.

A dialysis membrane (cellulose ester, 500–1000 Da molecular weight cut-off) was used as an ultrafilter for the as-prepared C-dots to separate the two surface states. As shown in Figure 3a, the 430 nm emission of the dialyzed C-dots that collected outside the membrane grew stronger after the first 2 h dialysis, but the 480 nm emission was weaker than that inside the membrane. Moreover, the UV absorption peak at approximately 360 nm for the dialyzed C-dots vanished, and this could be related to the weakness of the 480 nm emission. The fluorescence spectra of the C-dots dialyzed at various times are assembled in Figure S4, and the changes in the ratio of the PL intensity at 430 nm to that at 480 nm with the dialysis time are plotted in Figure 3b. The plot shows that the C-dot particles with higher ratio values penetrate through membrane faster than those with lower ratios, and each as-prepared C-dot particle did not have a uniform surface composition with an identical PL intensity ratio. The faster penetration was apparently not due to the smaller particle size because the average diameter (3.1 nm, σ = 1.0 nm) of the C-dots dialyzed for the first two hours was close to that (3.6 nm, σ = 1.1 nm) for those dialyzed for 8 h, as shown in Figure S5. The main reason for different penetration rate is that some of the functional groups or surface states leading to the 430 nm emission favored penetration, but other surface states that lead to the 480 nm emission did not favor penetration. These two groups simultaneously collected on a C-dot particle, but the buildup of the groups on the particles was in different mole ratios. Figure 3c shows a possible energy level diagram for a C-dot particle. For the two emissions, the excitation radiation beams actuated the valence π electrons in the core of the C-dot particle to the π* conduction band. Then, the excited electrons transitioned to the emissive surface states via radiationless relaxation between the lower π* levels and/or their hybrid states, which were hybridized with core carbon and heteroatoms, such as nitrogen and oxygen atoms, during the nucleation step in the carbonation. 

The upconversion emission at λ_em,max_ = 480 nm was observed for the C-dots with 710–790 nm excitation (λ_ex,max_ = 790 nm), as shown in Figure S6. The emission intensity increased with the excitation wavelength. A longer-wavelength light than 800 nm was not available due to the limitations of the fluorospectrophotometer used in this study, but a longer wavelength source, such as a near-IR laser, might induce a higher PL intensity. A 790 nm light stimulated the 480 nm up-conversion emission, and a 390 nm light also stimulated the 480 nm emission, as shown in Figure 1a. This was expected because 790 nm is nearly double 390 nm and is suitable for the sequential absorption of two long wavelength photons. However, a 430 nm up-conversion emission was not observed in the excitation range from 430 to 790 nm. The π* transit states (2λ = 660 nm) that correlated with the 430 nm emission at λ_ex,max_ = 330 nm, as shown in Figure 1d, would be less stable for the sequential absorption than those (2λ = 780 nm) that correlated with the 480 nm and underwent radiationless relaxation.

### 3.3. Comparison between CCP, Microwave, and Hydrothermal Carbonation

**Figure 4:**
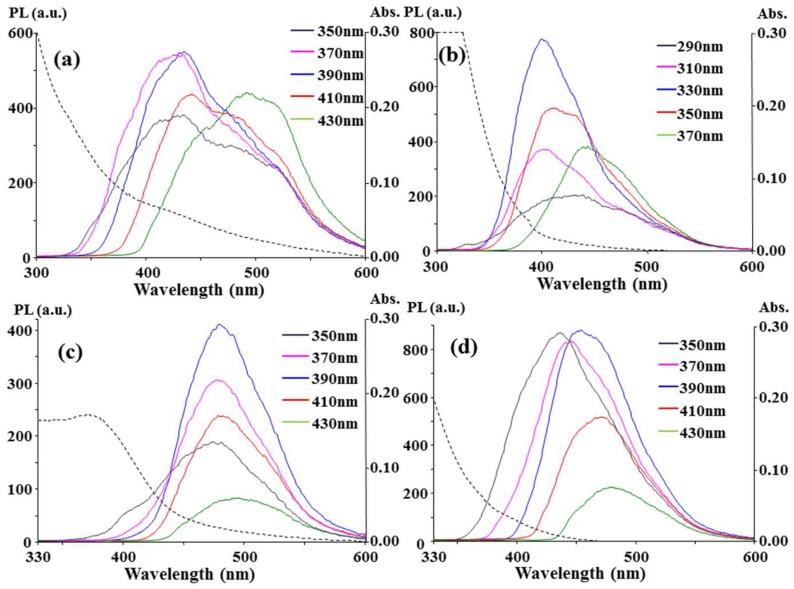
Fluorescence and UV-Vis absorption spectra for the CA + [EMIM]N(CN)_2_ (**a**,**c**) and [EMIM]N(CN)_2_ alone (**b**,**d**) samples heated in a 90 W microwave oven for 4.5 min (**a**,**b**) and in an autoclave at 200 °C for 30 min (**c**) and 5.0 h (**d**). After the addition of 1.0 mL of H_2_O to the heated products, the product solutions of 0.2 μL for (**a**); 0.3 μL for (**b**); 2.0 μL for (**c**); and 15 μL for (**d**) were diluted with 3.0 mL of H_2_O to measure the spectra.

For a comparison, CA and [EMIM]N(CN)_2_ were mixed in a crucible and heated in a domestic microwave oven at 90 Watt. The heating was stopped after 4.5 min because a severe burning flame appeared on the crucible during the microwave irradiation. As shown in Figure 4a, the microwave-treated product emission at 430 nm is stronger than its emission at 480 nm. Therefore, the number of surface states corresponding to the 430 nm emission should be larger than the number corresponding to the 480 nm emission. Furthermore, the chemical composition corresponding to the two surface states caused by microwave radiation with a sufficient oxygen supply should be different from that caused by the CCP ion bombardment under a limited oxygen supply. The stronger 430 nm emission obtained with a sufficient oxygen supply implied that the formation of the 430 nm emissive surface states involved oxygen atoms. Figure 4a shows the λ_ex,max_ was 370 nm or 390 nm for the maximum emission at λ_em,max_ = 430 nm, and the λ_ex,max_ was 430 nm for that at λ_em,max_ = 480 nm. The two λ_ex,max_ values are larger (approximately 40 nm) than those in Figure 1d (λ_ex,max_ = 330 nm) and Figure 1a (λ_ex,max_ = 390 nm), respectively. In addition to the changes in the surface composition, the severe carbonation in a microwave oven might extend the domain of the double bond and shrink the energy gap between π and π* to cause the 40 nm redshift compared with that for the CCP treatment. Without the addition of CA, heating [EMIM]N(CN)_2_ alone in a microwave could not provide the considerable 480 nm emission and only resulted in a strong 430 nm emission (λ_em,max_ = 400 nm) at a shorter λ_ex_ (290~350 nm), as shown in Figure 4b. In comparison with Figure 4a, the participation of CA in the carbonation could mainly contribute the 480 nm emissive states and the extension of the conjugated double bonds to the C-dot structure.

The mixture of CA and [EMIM]N(CN)_2_ was also placed in a Teflon-lined autoclave and heated in a hot-air oven at 200 °C for 30 min. The PL and UV-Vis absorption spectra of the hydrothermal products are plotted in Figure 4c and are similar to those in Figure 1a, which were obtained from O_2_/CCP at 10 sccm, except the PL intensity was smaller than that in Figure 1a. Furthermore, the spectrum of the [EMIM]N(CN)_2_ alone sample treated by a hydrothermal method was similar to that from the CCP method, i.e., comparison of Figure 4d with Figure 1b. It is believed that fine tuning the temperature and duration of the hydrothermal reaction in an autoclave could enhance the similarity of the reaction to the CCP carbonation in a vacuum chamber with a low O_2_ supply, i.e., 10 sccm in our system. Figure 4d shows the results from 5.0 h of heating [EMIM]N(CN)_2_ alone in an autoclave. Only 30 min of heating by the hydrothermal method could not obtain a PL spectrum, but Figure 1b spectrum by the O_2_/CCP method was obtained.

### 3.4. Fluorescent Sensing by the O_2_/CCP-Treated C-Dots

**Figure 5:**
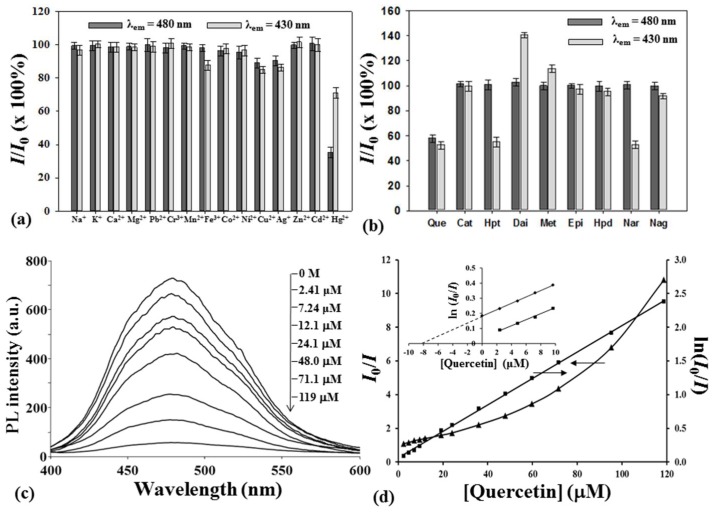
Effect of different metal ions (33.3 μM) (**a**) and flavonoids (8.33 μg/mL) (**b**) on the relative PL intensity (*I*/*I*_0_) of the C-dots prepared in the conditions of Figure 1a; (**c**): Emission spectra of the prepared C-dots before and after the addition of various concentrations of quercetin; (**d**): The respective emission intensities are plotted versus the quercetin concentrations according to the Stern–Volmer (▲) and Perrin (■) models. The curve of the standard addition calibration (♦) for the determination of quercetin in the ethanol extract of Guang-Chen-Pi is inserted in (**d**). I and I_0_ are the PL intensities observed in the presence (*I*) and absence (*I*_0_), respectively, of the samples in a 50 mM phosphate buffer at pH 7.0.

The fluorescent C-dots prepared by O_2_/CCP of a mixture of CA and [EMIM]N(CN)_2_ in the conditions shown in the legend of Figure 1a were used to separately probe fifteen metal ions and nine flavonoids. At first, the λ_ex_-independent emissions at 430 nm (λ_ex,max_ = 330 nm) and 480 nm (λ_ex,max_ = 390 nm) of the C-dots were used to evaluate their responses to the analytes. The 480 nm emission was quenched by Cu^2+^, Ag^+^, and Hg^2+^, while the 430 nm emission was further quenched by Fe^3+^ in addition to those ions, as shown in Figure 5a, where *I* and *I*_0_ are the emission intensities in the absence and presence of the sample, respectively. The ions, Fe^3+^, Cu^2+^, and Ag^+^, quenched 10~15% of each emission, and nearly 30% of the 430 nm emission and 65% of the 480 nm emission could be heavily quenched by Hg^2+^. The quenching was sensitive to Hg^2+^, especially the 480 nm emission, but the coexistence of the Fe^3+^, Cu^2+^, and Ag^+^ ions in a real sample would interfere with the detection of Hg^2+^. However, the synthetic C-dots are good starting fluorescent materials for further ligand attachment on them to improve a sensor’s specificity for Hg^2+^ in the presence of interfering ions.

For the flavonoid samples, quercetin, hesperetin, and naringenin could quench nearly half of the 430 nm emission, but daidzein and 5-methoxyflavone passivated some unknown surface traps and enhanced the 430 nm PL intensity, as shown in Figure 5b. In contrast to the 430 nm emission, the 480 nm emission was only selectively quenched by quercetin, and the other flavonoids did not affect the 480 nm emission. Therefore, the C-dots would directly sense quercetin in a flavonoid-rich sample, such as *Citrus reticulata* cv. Chachiensis, which is a sun-dried peel used as a traditional Chinese medicine, called “Guang-Chen-Pi” in Chinese. For quantification, the 480 nm PL intensities were measured (Figure 5c) after equilibrium with quercetin standards in a phosphate buffer, pH 7.0, 50 mM, and they were plotted against the quercetin concentrations ranging from 2.4 μM to 119 μM in Figure 5d. As shown in Figure 5d, the Stern–Volmer plot, *I*_0_/*I* v.s. [quercetin], is not linear. The deviation from linearity is frequently attributed to a combination of dynamic and static quenching and can be corrected using a modified Stern–Volmer plot, i.e., the Perrin model:

ln (*I*_0_/*I*) = *N*_A_*V* [Quencher]

where α = *N*_A_*V*, where *N*_A_ is Avogadro’s number and *V* is the volume of the active sphere of quenching. Based on the good linearity (*R*^2^ = 0.9992) and high slope (1.96 × 10^4^ M^−1^) of the Perrin relationship, the radius of the effective quenching sphere was calculated to be 19.8 nm, which is 2.3 times the C-dot radius (8.6 nm) and allowed an efficient, photoinduced electron-transfer process of the encounter pair between the C-dot (as an electron donor) and quercetin (as an electron acceptor) without coupling reagents. The relative standard deviation for seven replicate measurements of 12.1 μM quercetin solutions was 3.3%. Based on the 3σ of the blank response (σ = 3.1%, *n* = 10), the detection limit was calculated to be 0.5 μM. As shown in the inset of Figure 5d, the quercetin in the ethanol extract of Guang-Chen-Pi was determined by a standard addition method to be 4.20 ± 0.15 mg/g, which was nearly nine times higher than 0.47 mg/g found in the air-dried peel of *Citrus reticulate* Blanco [29]. Some factors, such as the citrus species used, growth environment, and method of drying the peel, accounted for the difference. The matrix effect was evaluated by the ratio defined as (*S*_1_ − *S*_2_) × 100%/*S*_2_, where *S*_1_ and *S*_2_ are the slopes of the calibration curves obtained by standard addition (*S*_1_ = 2.12 × 10^4^ M^−1^, *R*^2^ = 0.9990) and external standard (*S*_2_ = 1.96 × 10^4^ M^−1^) methods, respectively [30]. The calculated ratio, 8.16%, was lower than 10%, which suggested that the matrix effect could be ignored by the standard addition method. The selectivity of the 480 nm emission from the C-dots for quercetin helped reduce the matrix effect.

## 4. Conclusions

The use of O_2_/CCP in the carbonation of mixtures of aliphatic acids and nitrogen-containing viscous liquids, such as ionic liquids and PEI, was successful in forming fluorescent molecules. The dispersion of citric acid in [EMIM]N(CN)_2_ possessed the highest fluorescence intensity among the mixtures, and the characteristics of the carbon quantum dots with C–N, C–O, O–H, and N–H functionalities were two excitation-independent emissions emerging from two surface states and upconversion of the fluorescence. In a comparison of the fluorescence spectra, the CCP-treated C-dots were similar to the hydrothermally synthesized ones because they were operated in an oxygen-limited environment, but they were different from the MW-irradiated ones in an open environment. With specificity for quercetin among nine flavonoids, the CCP-treated C-dots were applied to determine the quercetin content in Guang-Chen-Pi, 4.20 ± 0.15 mg/g. Among fifteen metal ions, mercury ions most effectively quenched the fluorescence of the CCP-treated C-dots. Instead of O_2_/CCP, the N_2_/CCP treatment of citric acid in glycerol under the same CCP conditions converted non-fluorescent C-dots to fluorescent ones. This CCP-gas system can, not only provide carbonization of carbonaceous materials but doping of heteroatoms or afterwards.

## Supplementary Materials

The following are available online at http://www.mdpi.com/2079-4991/8/6/372/s1, Figure S1: TEM images of the as-prepared C-dots; Figure S2: The deconvolution of the C 1s, N 1s, and O 1s XPS peaks from the O_2_/CCP of the mixture of CA and [EMIM]N(CN)_2_; Figure S3: Plot of the normalized PL intensity against the UV-Vis absorbance for the determination of the quantum yield; Figure S4: The fluorescence spectra of the C-dots collected after different dialysis times; Figure S5: TEM images of the C-dots dialyzed for two hours and their size distribution; Figure S6: Up-conversion emissions of the prepared C-dots under different wavelength excitations; Table S1: The UV-Vis and fluorescence spectra of the products obtained by CCP treatments.
