# Supplementary material for: Capacitively Coupled Plasma Discharge of Ionic Liquid Solutions to Synthesize Carbon Dots as Fluorescent Sensors
Source: Nanomaterials (Basel). 2018 May 26;8(6):372. doi: 10.3390/nano8060372 (PMC6027164; doi:10.3390/nano8060372)

Electronic Supplementary Material

Capacitively Coupled Plasma Discharge of Ionic Liquid Solutions to Synthesize Carbon Dots as Fluorescent Sensors

Table S-1

The UV-Vis and fluorescence spectra of the products obtained by CCP treatments (O_2: 10 sccm, RF: 90 W, 30 min) of mixtures of different aliphatic acids (100 mg) and high b.p. solvents (500 µL). After CCP treatment, 1.0 mL of H_2O was used to dissolve the treated products. If not, there is a note in the first column. Different aliquots (µL) of the product solutions were diluted to 3.0 mL by H_2O and are specified in the rows below the UV-Vis spectra. The diluted products were measured by UV-Vis and fluorescence spectroscopy, and their maximum fluorescent intensities (I_{max} (a.u.)) at excitation ($\lambda_{ex,max}$ (nm)) and emission ($\lambda_{em,max}$ (nm)) wavelengths are recorded on the same rows. Different colored lines shown in the fluorescence spectra represent the excitation at different wavelengths. Δ λ (nm) denotes the emission redshift wavelength, which was the difference in the emission wavelength between the excitations at the longest and shortest wavelengths in the fluorescence spectra.

	**Citric acid** 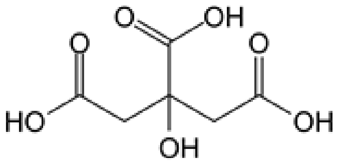	**Succinic acid** 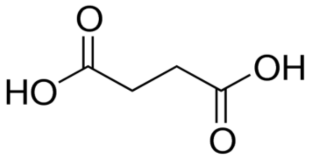	**Adipic acid** 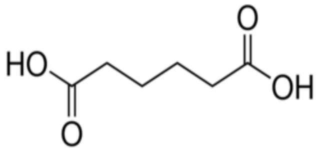	**Malic acid** 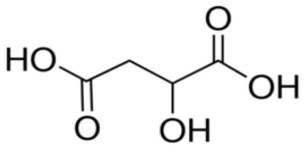
**Glycerol** 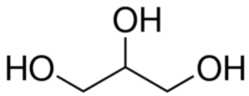	**X**	**X**	**X**	**X**
	**20 µL**	**20 µL**	**20 µL**	**20 µL**

**Poly(ethylene glycol)** 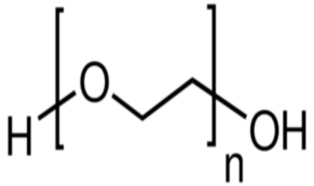 **(PEG 1000)**	**X**	**X**	**X**	**X**
	**20 µL**	**20 µL**	**20 µL**	**20 µL**
**Polyethylenimine, branched** 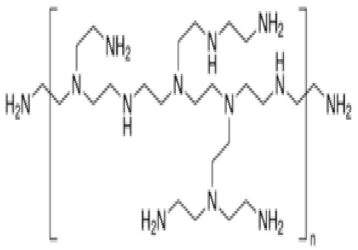 **(PEI 600)**	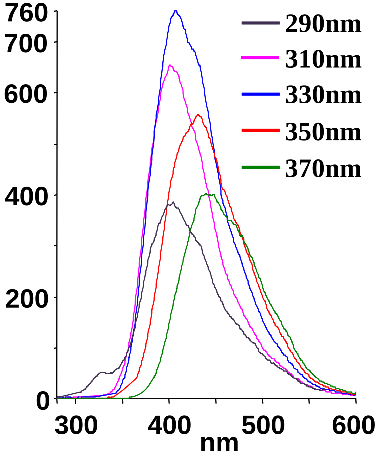 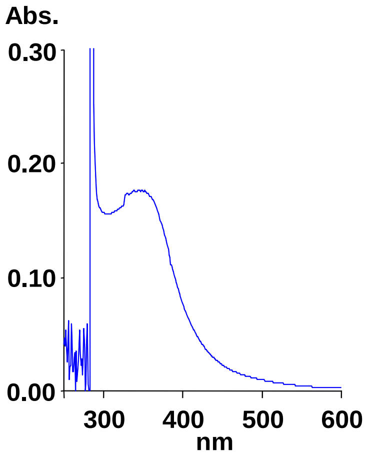	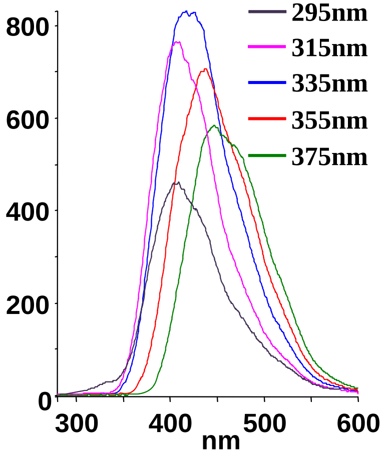 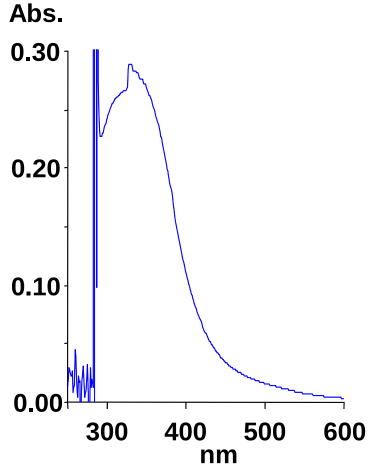	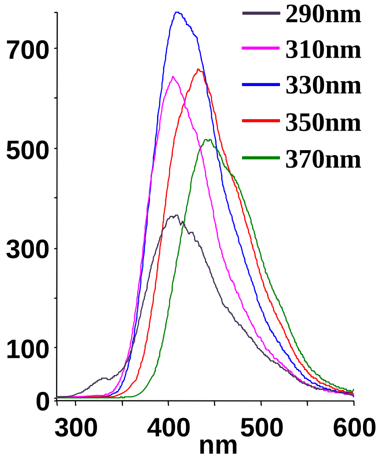 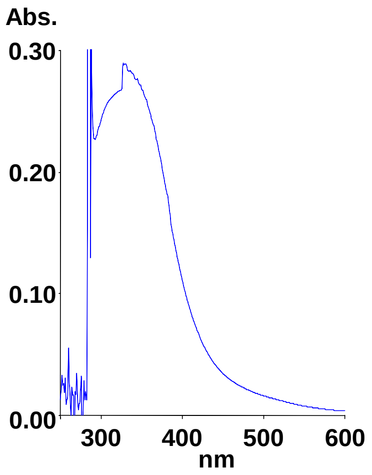	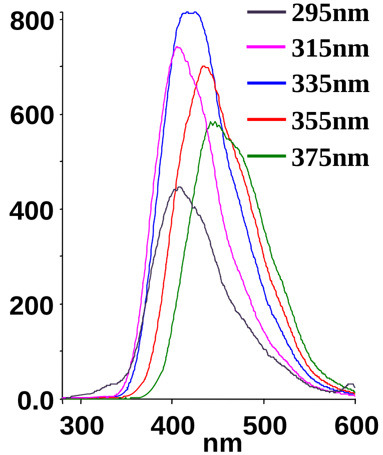 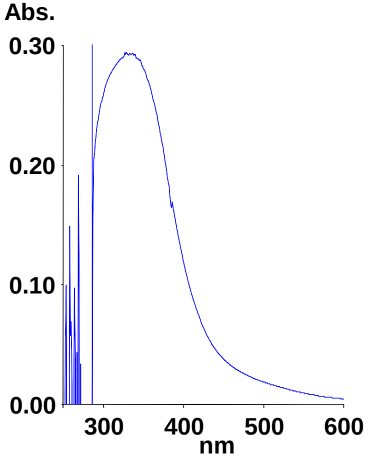
	$\lambda_{ex,max}$ 330 nm, $\lambda_{em,max}$ 408 nm,	$\lambda_{ex,max}$ 335 nm, $\lambda_{em,max}$ 416 nm,	$\lambda_{ex,max}$ 330 nm, $\lambda_{em,max}$ 410 nm,	$\lambda_{ex,max}$ 335 nm, $\lambda_{em,max}$ 419 nm,

<table>
<tr><th></th><th>I = 760, 20 μL, Δλ = 40 nm</th><th>I = 829, 20 μL, Δλ = 40 nm</th><th>I = 770, 20 μL, Δλ = 38 nm</th><th>I = 813, 20 μL, Δλ = 39 nm</th></tr>
<tr>
<td rowspan="2">1-Ethyl-3-methyl-imidazolium dicyanamide
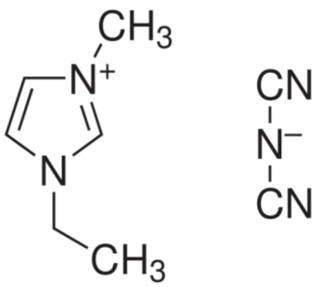

([EMIM]N(CN)2)</td>
<td>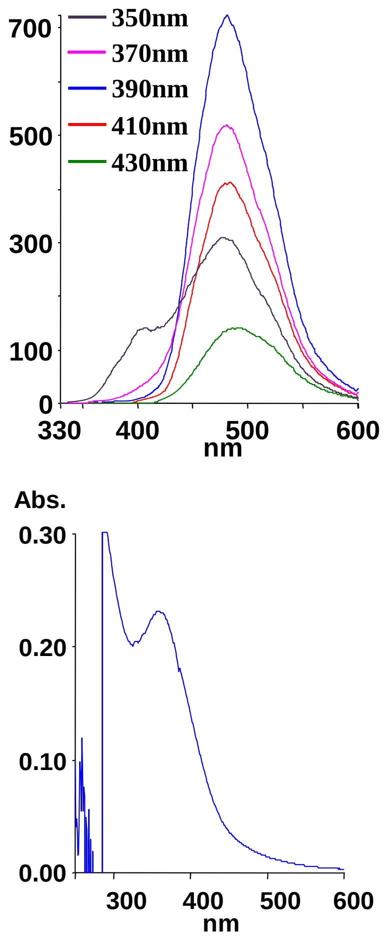</td>
<td>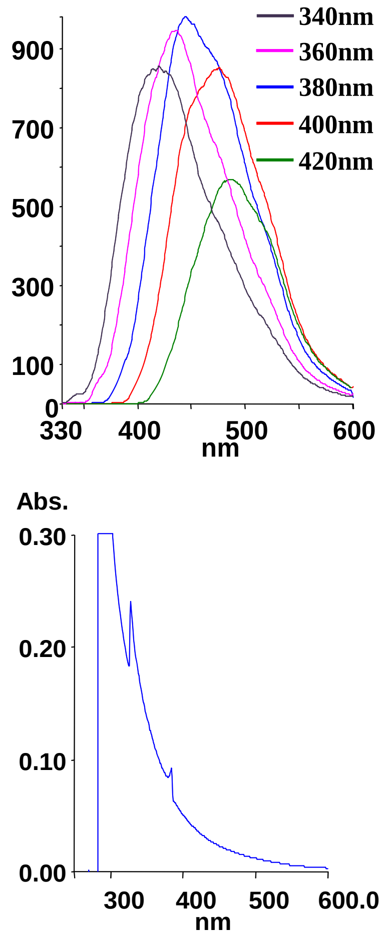</td>
<td>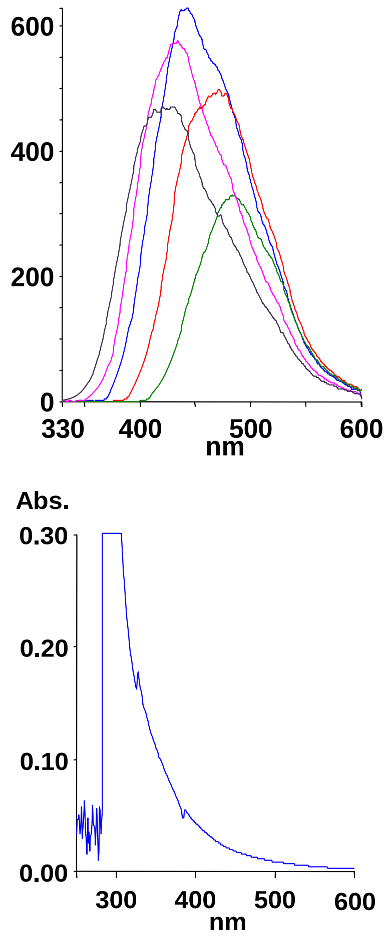</td>
<td>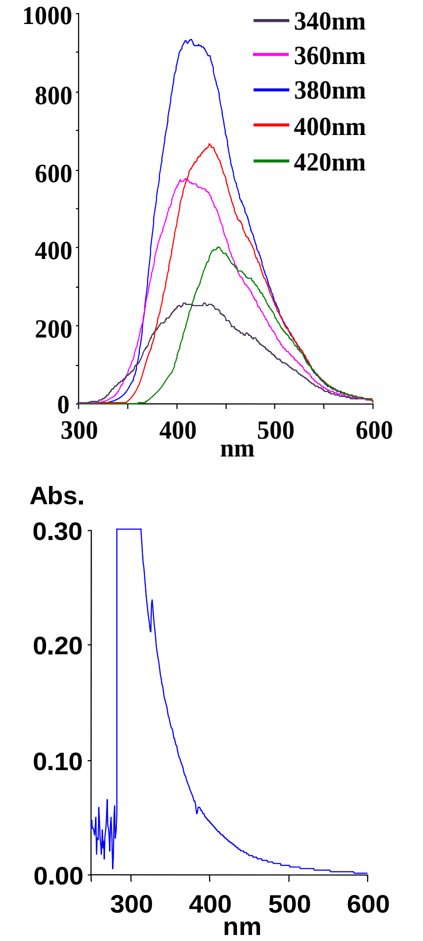</td>
</tr>
<tr>
<td>$\lambda_{ex,max}$ 390 nm, Em 482 nm,
I = 723, 0.2 μL, Δλ = 11 nm</td>
<td>$\lambda_{ex,max}$ 380 nm, $\lambda_{em,max}$ 444 nm,
I = 982, 20 μL, Δλ = 66 nm</td>
<td>$\lambda_{ex,max}$ 380 nm, $\lambda_{em,max}$ 441 nm,
I = 625, 20 μL, Δλ = 60 nm</td>
<td>$\lambda_{ex,max}$ 340 nm, $\lambda_{em,max}$ 406 nm,
I = 934, 4 μL, Δλ = 7 nm</td>
</tr>
</table>

<table>
<tr>
<td rowspan="2">1-Ethyl-3-methyl-imidazolium tetrachloroaluminate
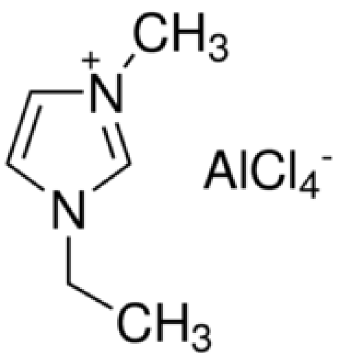

([EMIM]AlCl$_4$)</td>
<td>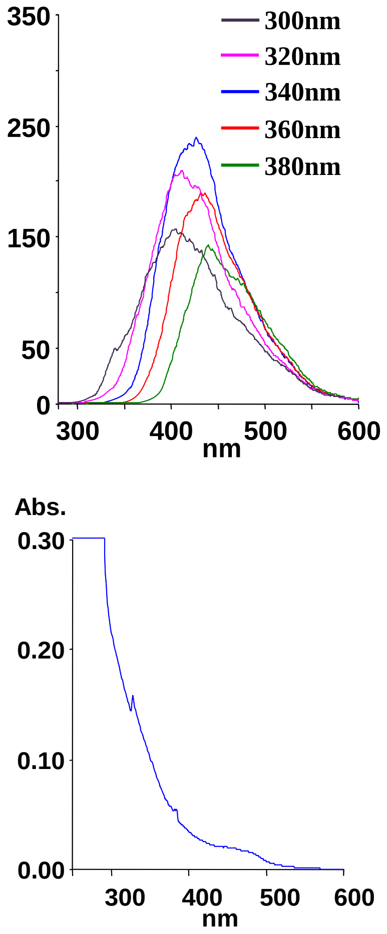
</td>
<td>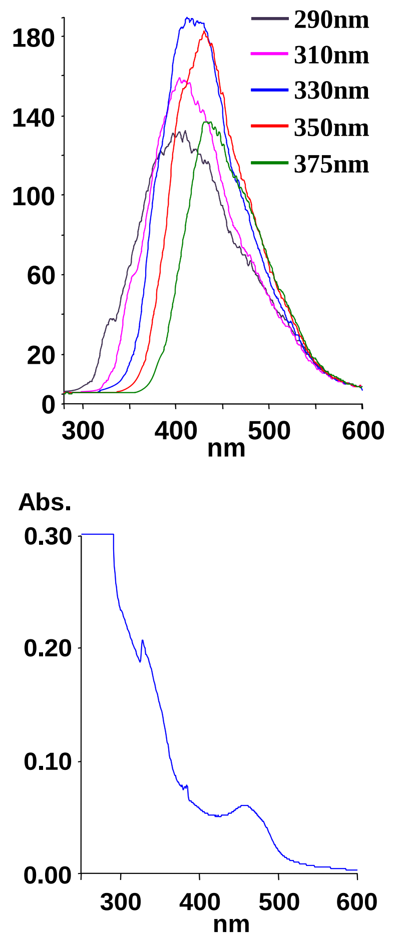
</td>
<td>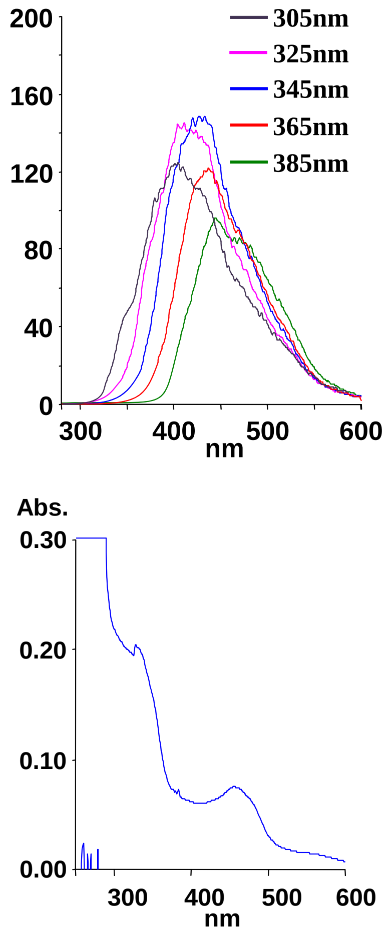
</td>
<td>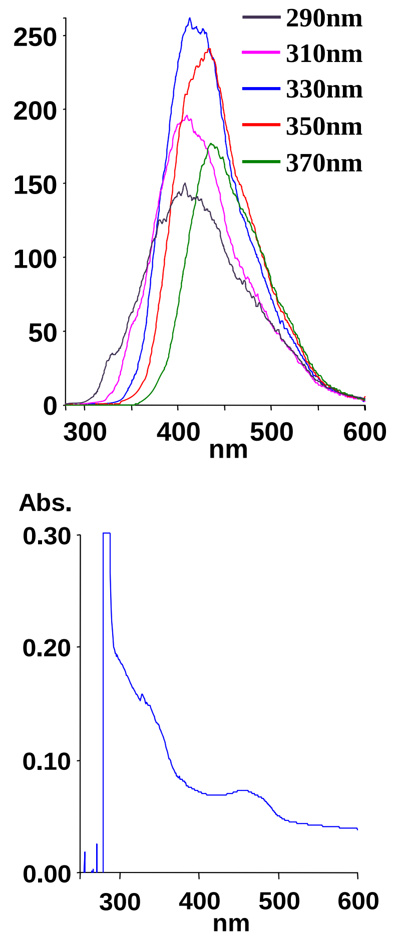
</td>
</tr>
<tr>
<td>$\lambda_{ex,max}$ 340 nm, $\lambda_{em,max}$ 427 nm, I = 240, 20 μL, $\Delta\lambda$ = 29 nm</td>
<td>$\lambda_{ex,max}$ 330 nm, $\lambda_{em,max}$ 418 nm, I = 188, 20 μL, $\Delta\lambda$ = 33 nm</td>
<td>$\lambda_{ex,max}$ 345 nm, $\lambda_{em,max}$ 429 nm, I = 148, 20 μL, $\Delta\lambda$ = 44 nm</td>
<td>$\lambda_{ex,max}$ 330 nm, $\lambda_{em,max}$ 411 nm, I = 260, 20 μL, $\Delta\lambda$ = 28 nm</td>
</tr>
</table>

1-Butyl-3-methyl-imidazolium tetrafluoroborate

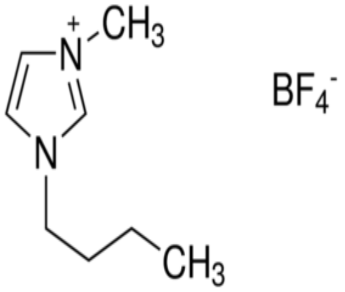

([BMIM]BF_4)

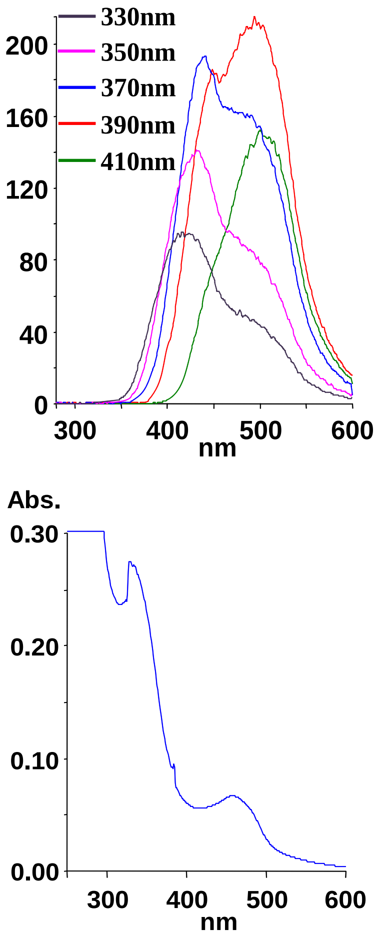

$\lambda_{ex,max}$ 390 nm, $\lambda_{em,max}$ 497 nm, I = 211, 20 μL, Δλ = 82 nm

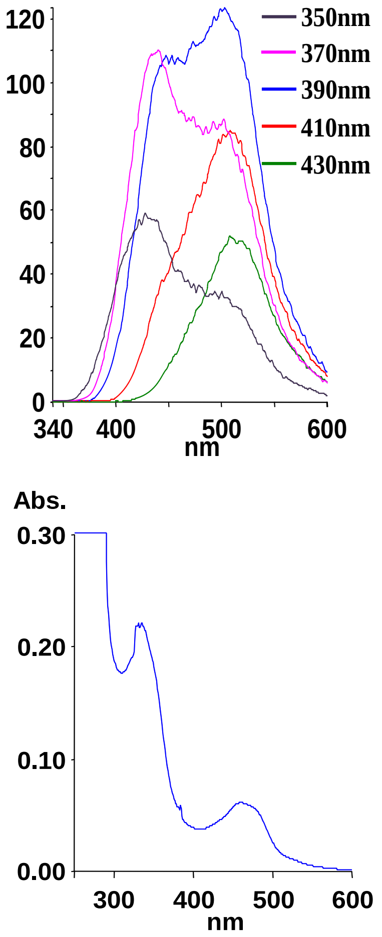

$\lambda_{ex,max}$ 390 nm, $\lambda_{em,max}$ 503 nm, I = 123, 20 μL, Δλ = 82 nm

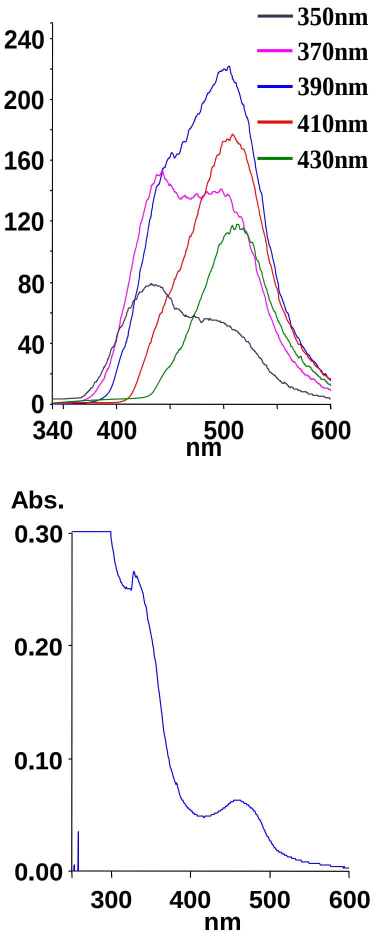

$\lambda_{ex,max}$ 390 nm, $\lambda_{em,max}$ 502 nm, I = 219, 20 μL, Δλ = 79 nm

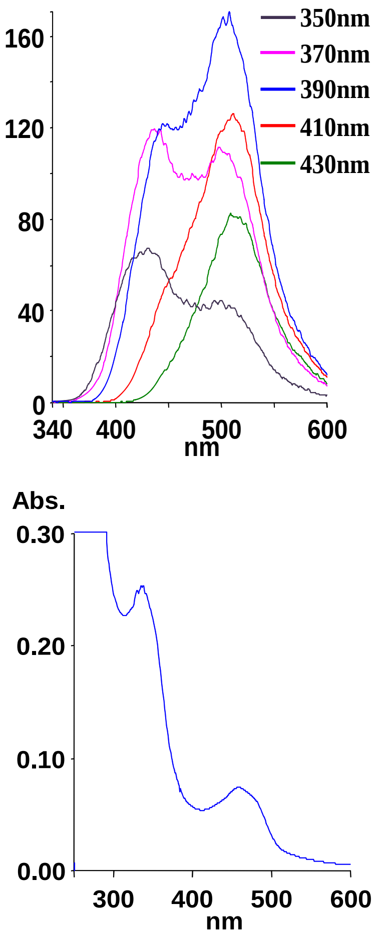

$\lambda_{ex,max}$ 390 nm, $\lambda_{em,max}$ 504 nm, I = 165, 20 μL, Δλ = 81 nm

1-Butyl-3-methyl-imidazolium chloride

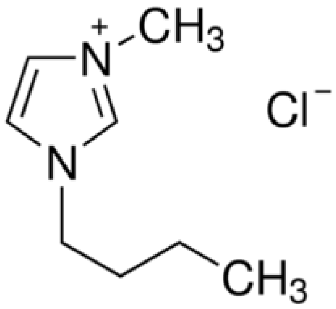

([BMIM]Cl])

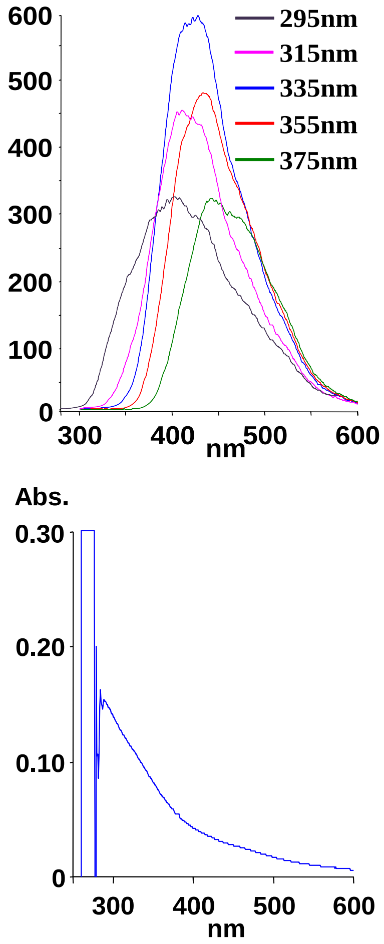

$\lambda_{ex,max}$ 335 nm, $\lambda_{em,max}$ 422 nm, I = 584, 20 μL, Δλ = 33 nm

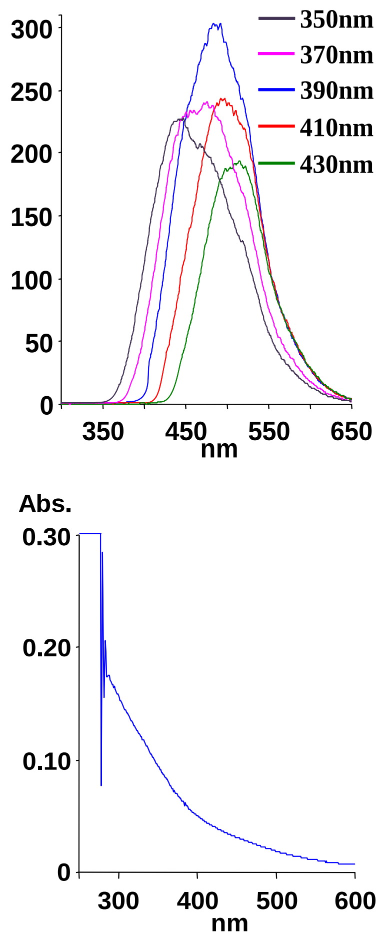

$\lambda_{ex,max}$ 390 nm, $\lambda_{em,max}$ 485 nm, I = 302, 20 μL, Δλ = 52 nm

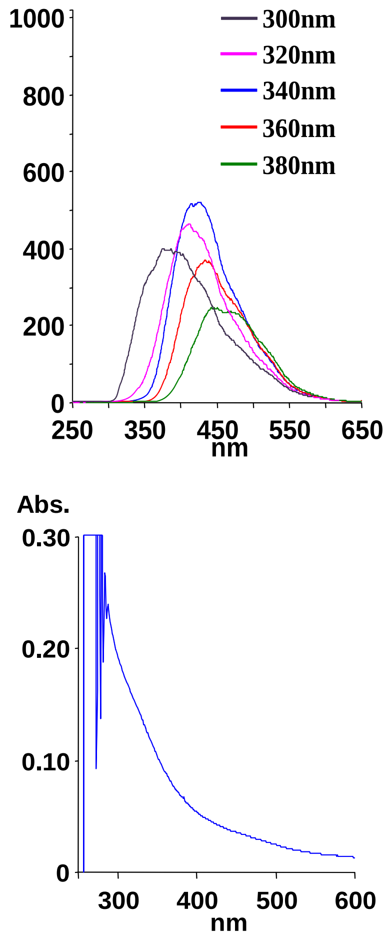

$\lambda_{ex,max}$ 340 nm, $\lambda_{em,max}$ 425 nm, I = 520, 20 μL, Δλ = 63 nm

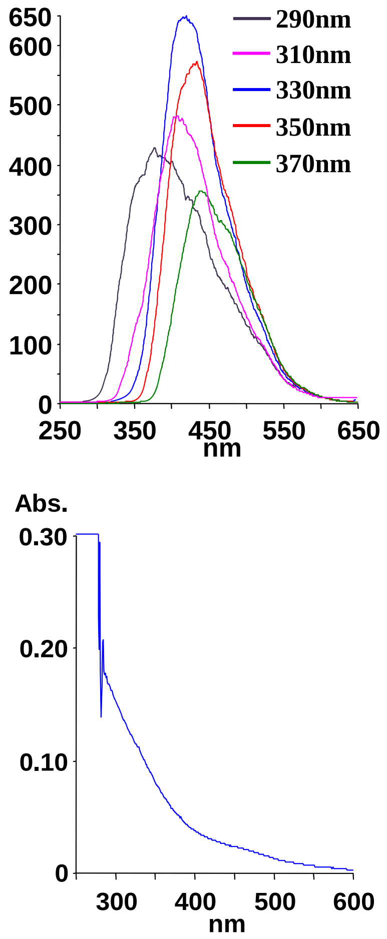

$\lambda_{ex,max}$ 330 nm, $\lambda_{em,max}$ 420 nm, I = 648, 20 μL, Δλ = 62 nm

1-Butyl-3-methyl-imidazolium bromide

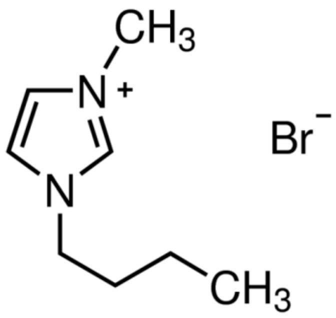

([BMIM]Br)

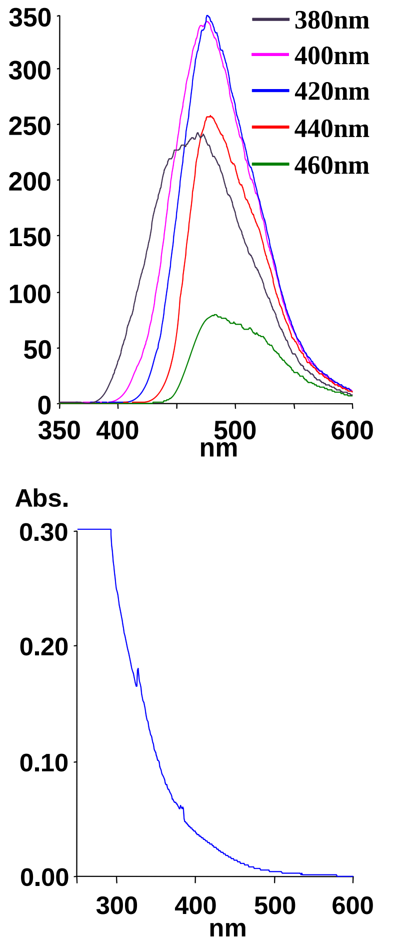

$\lambda_{ex,max}$ 420nm, $\lambda_{em,max}$ 498 nm, I = 347, 20 μL, Δλ = 7 nm

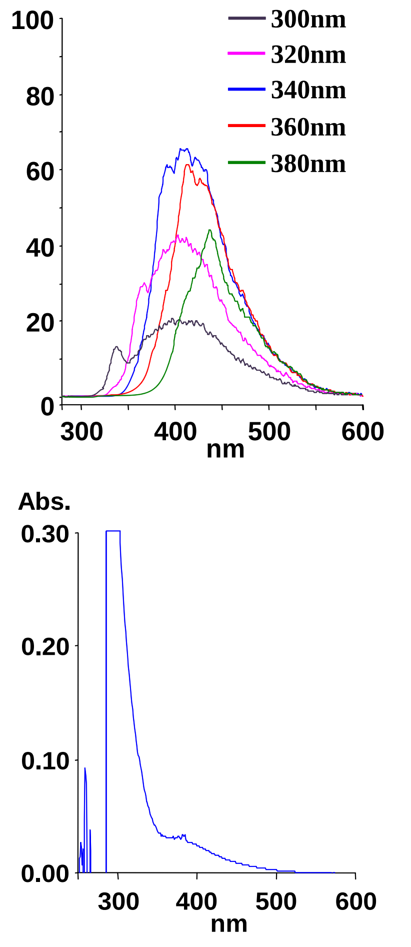

$\lambda_{ex,max}$ 340 nm, $\lambda_{em,max}$ 409 nm, I = 65, 20 μL, Δλ = 34 nm

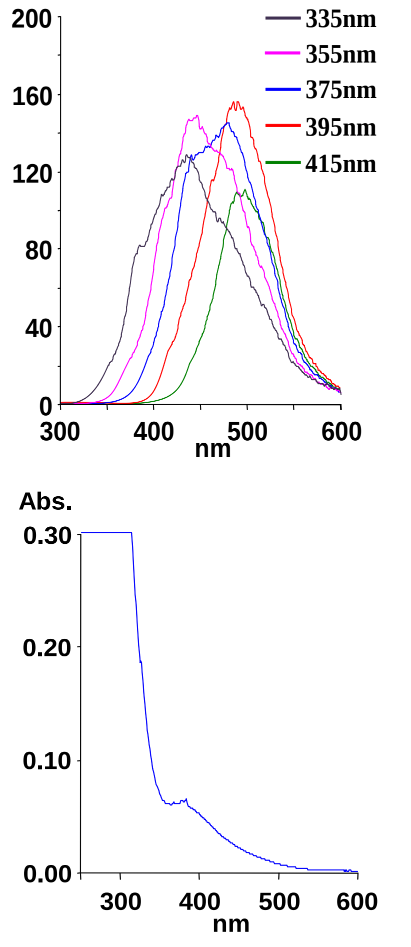

$\lambda_{ex,max}$ 395 nm, $\lambda_{em,max}$ 488 nm, I = 153, 20 μL, Δλ = 57 nm

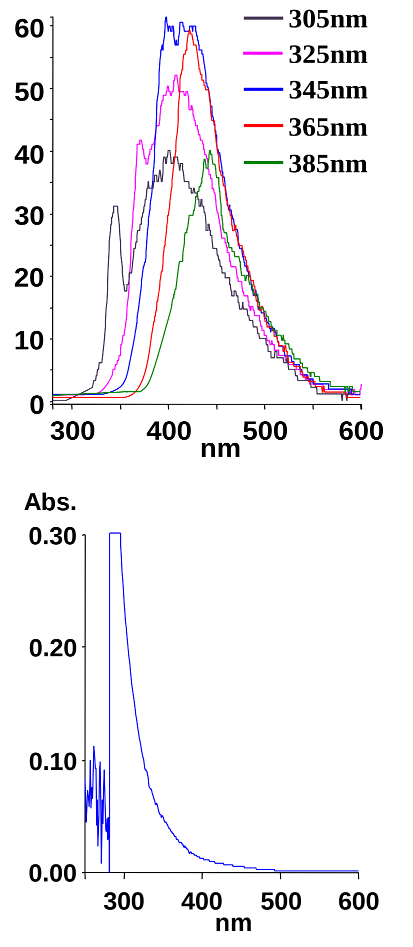

$\lambda_{ex,max}$ 345 nm, $\lambda_{em,max}$ 414 nm, I = 63, 20 μL, Δλ = 44 nm

1-Methyl-3-octyl-imidazolium chloride<br>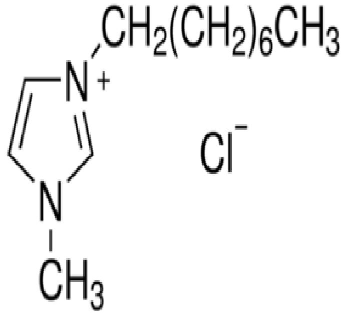<br><br>([MOIM]Cl)	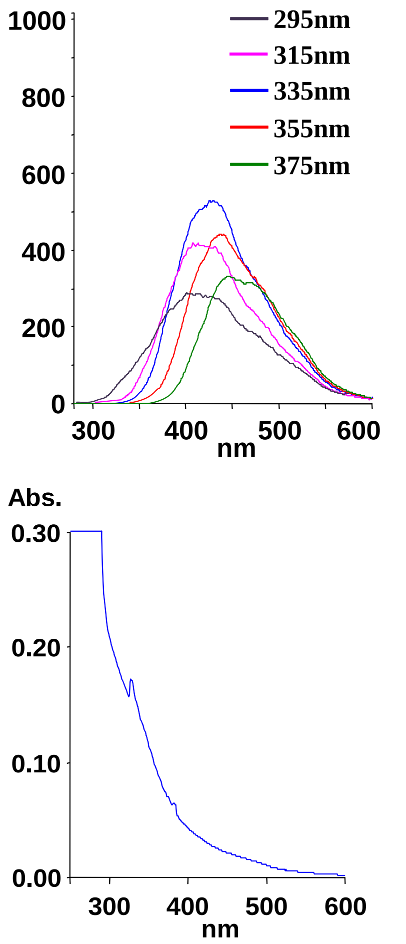 	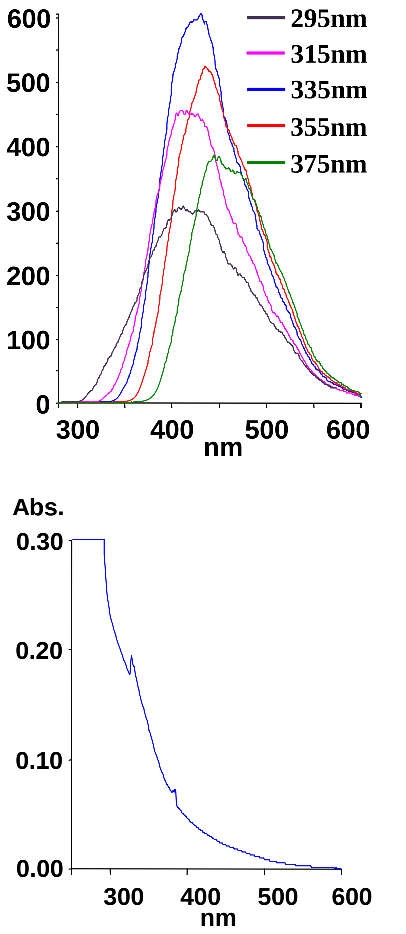 	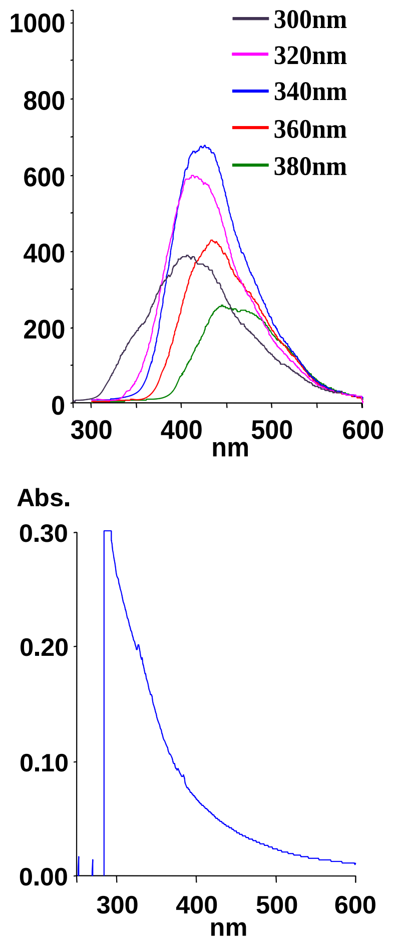 	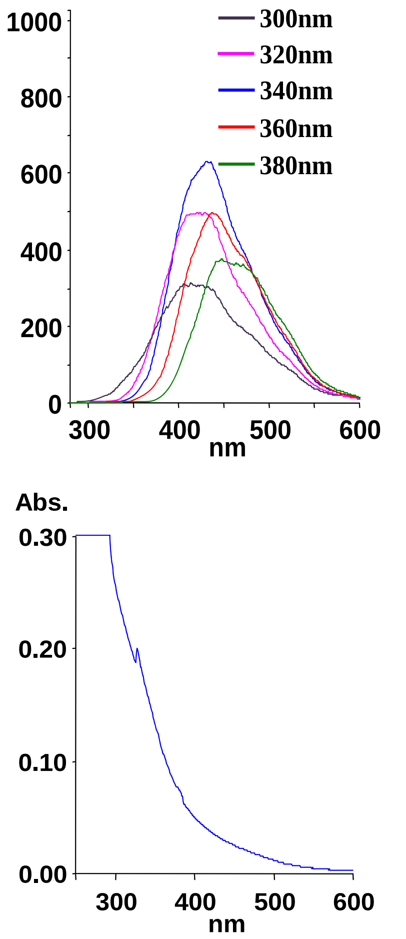
	$\lambda_{ex,max}$ 335 nm, $\lambda_{em,max}$ 427 nm, I = 525, 20 μL, Δλ = 40 nm	$\lambda_{ex,max}$ 335 nm, $\lambda_{em,max}$ 427 nm, I = 597, 20 μL, Δλ = 27 nm	$\lambda_{ex,max}$ 340 nm, $\lambda_{em,max}$ 421 nm, I = 675, 20 μL, Δλ = 32 nm	$\lambda_{ex,max}$ 340 nm, $\lambda_{em,max}$ 433 nm, I = 628, 20 μL, Δλ = 12 nm

Trihexyltetradecyl-phosphonium dicyanamide

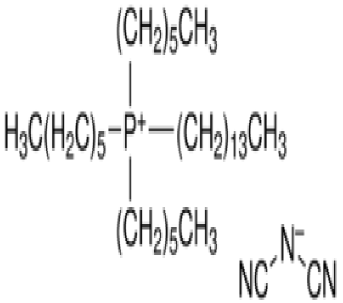

([P6,6,6,14]N(CN)$_2$)

Note: 2.0 mLEtOH replaced 1.0 mL H_2O to dissolve the product after CCP treatment

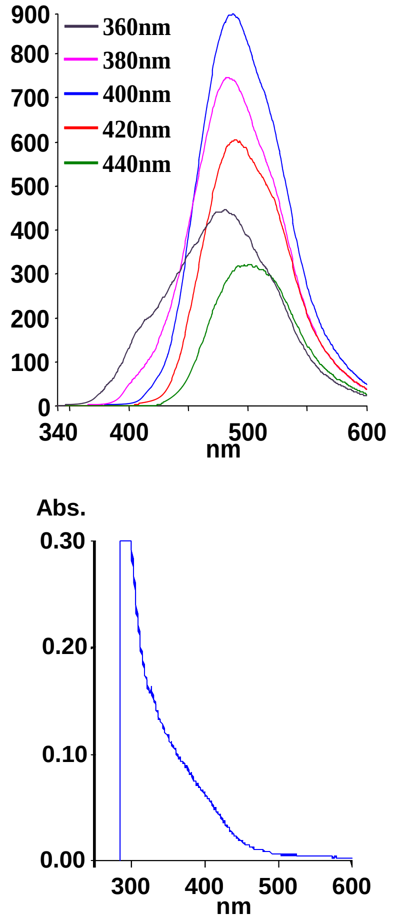

$\lambda_{ex,max}$ 400 nm, $\lambda_{em,max}$ 487 nm, I = 890, 5 µL, Δλ = 17 nm

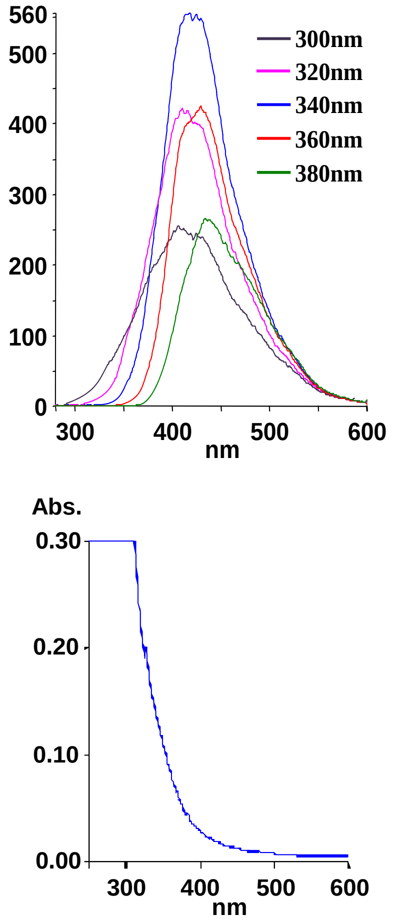

$\lambda_{ex,max}$ 340 nm, $\lambda_{em,max}$ 419 nm, I = 555, 20 µL, Δλ = 22 nm

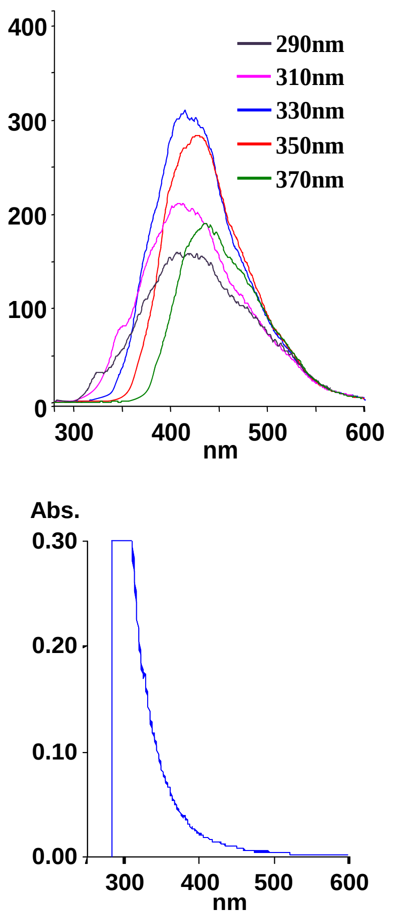

$\lambda_{ex,max}$ 330 nm, $\lambda_{em,max}$ 415 nm, I = 309, 20 µL, Δλ = 25 nm

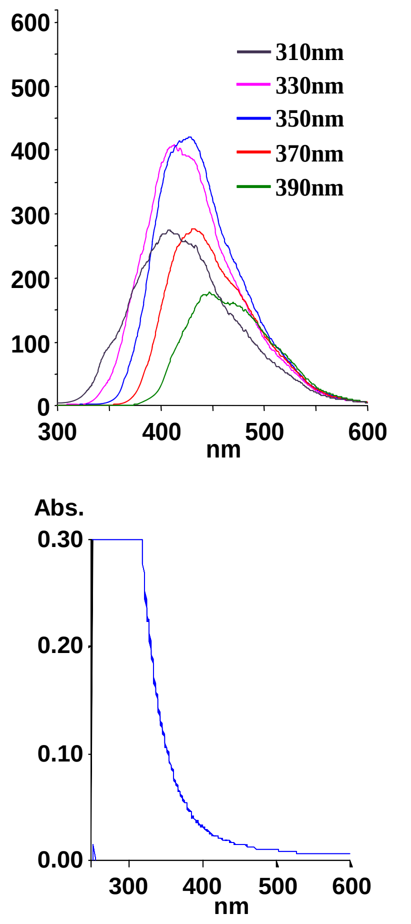

$\lambda_{ex,max}$ 350 nm, $\lambda_{em,max}$ 428 nm, I = 421, 20 µL, Δλ = 40 nm

Fig. S-1 TEM images of the as-prepared C-dots. (a) the original image input into the ImageJ software, (b) setting an appropriate threshold parameter in ImageJ to define the particle position, (c) circling the area of each particle using ImageJ, (d) histogram of the particle size distribution, (e) lattice fringe.

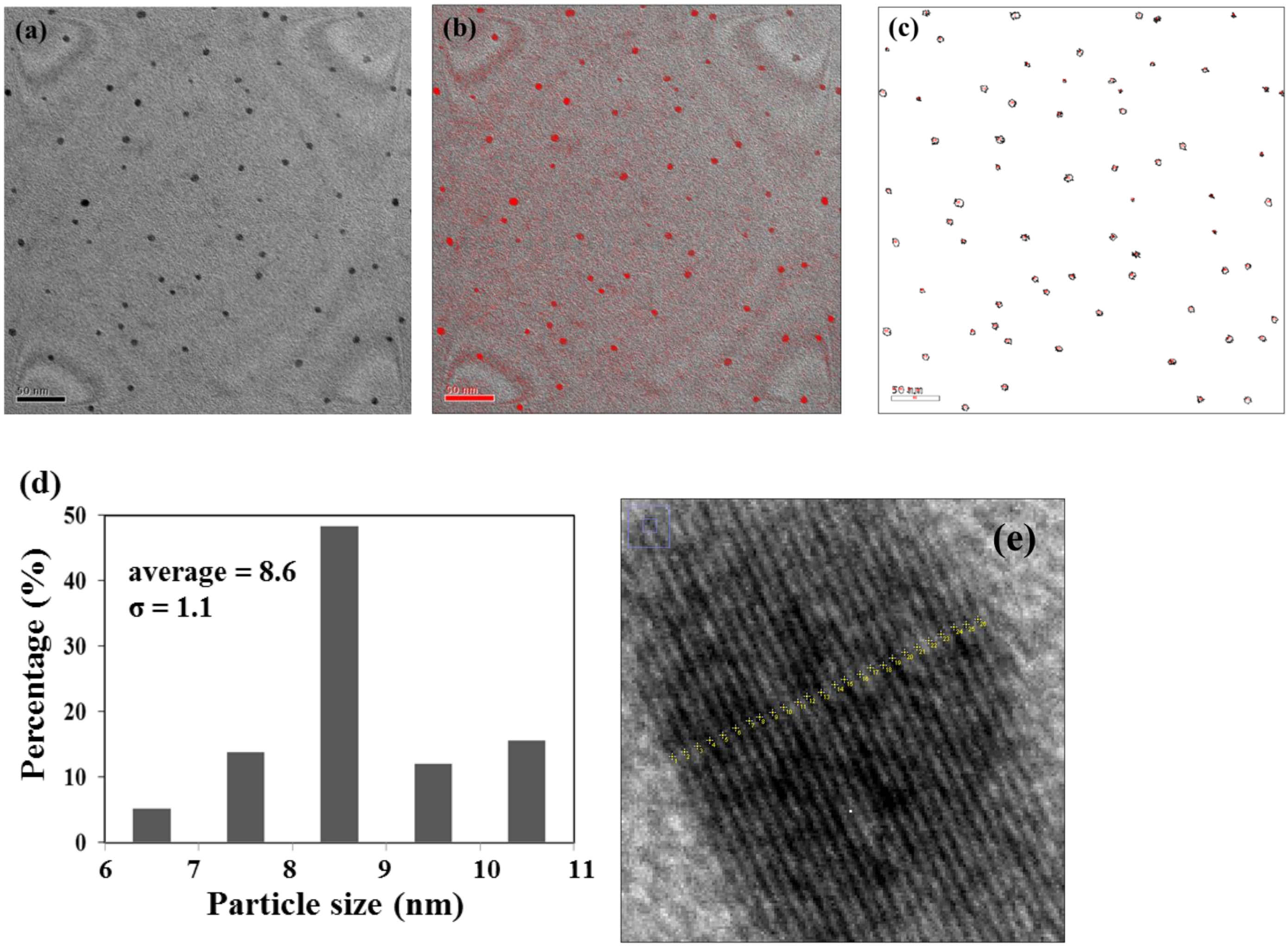

Fig. S-2 The deconvolution of the C 1s, N 1s, and O 1s XPS peaks from the O_2/CCP of the mixture of CA and [EMIM]N(CN)$_2$.

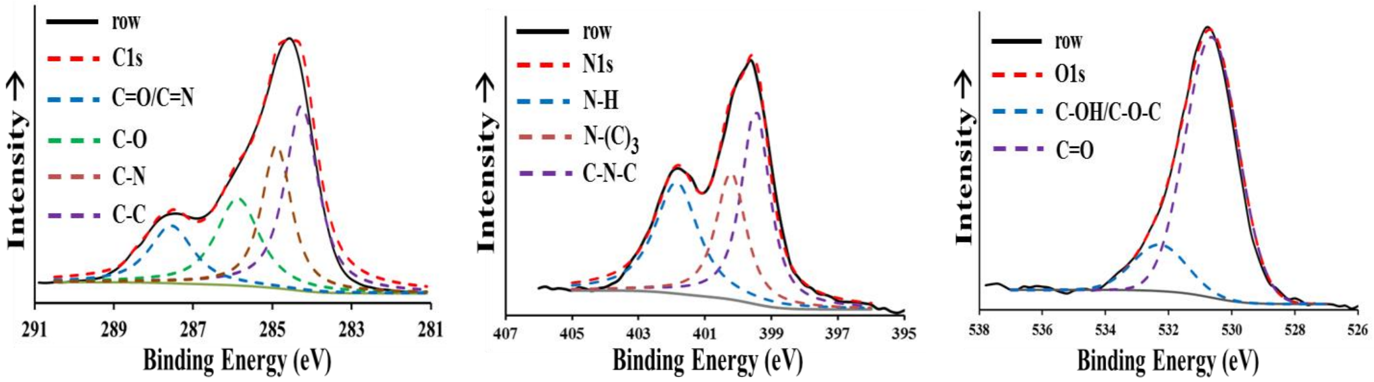

Fig. S-3 Plot of the normalized PL intensity against the UV-Vis absorbance for the determination of the quantum yield.

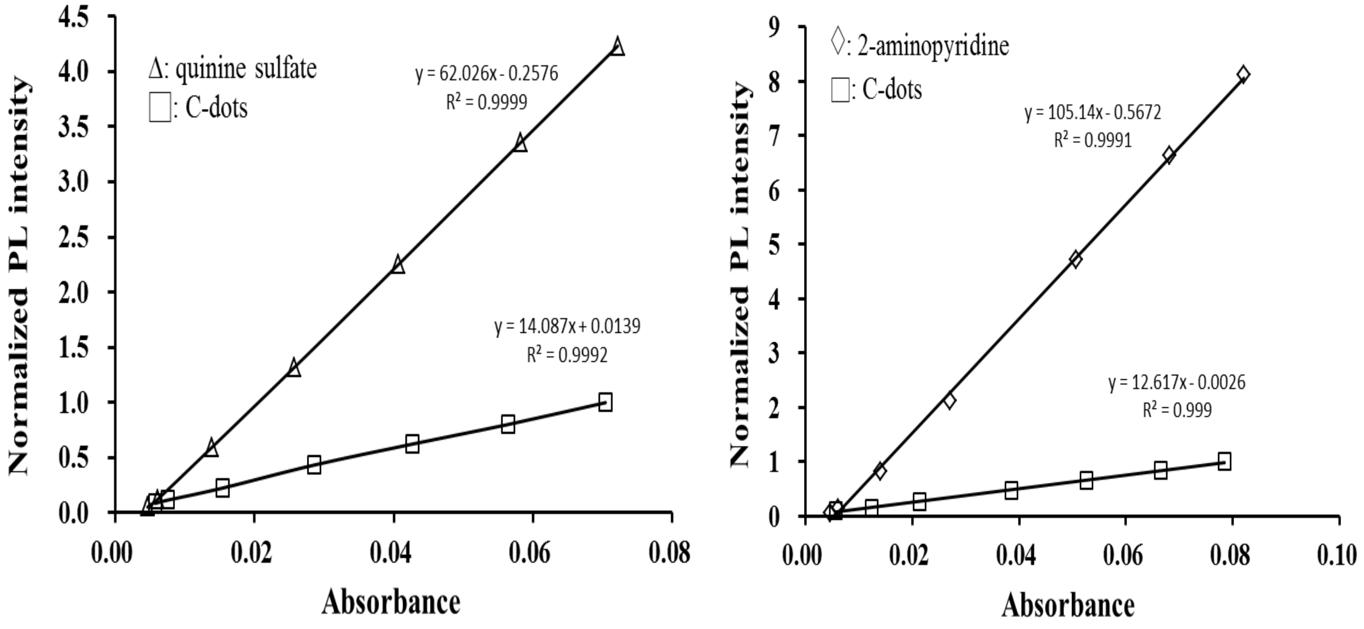

According to the slope method [1], the quantum yield (QY, Φ) could be determined by the following equation.

$\Phi_x = \Phi_{ref}\ (S_x / S_{ref})\ (\eta_x^2 / \eta_{ref}^2)$

where x, ref, S, and η denote the sample solution, reference solution, slope of the plot of the measured integrated emission intensity against the optical density, and refractive index of the solvent, respectively. Quinine sulfate (QY = 54.6%) and 2-aminopyridine (QY = 60.0%) in 0.1 M H_2SO4 were chosen as the references for the $\lambda_{em,max}$ = 480 nm ($\lambda_{ex,max}$ = 390 nm) and $\lambda_{em,max}$ = 430 nm ($\lambda_{ex,max}$ = 330 nm) emissions, respectively, of the studied C-dots [2]. To minimize the reabsorption effects, the absorbencies were kept under 0.1 at the excitation wavelengths. Both the η_x and η_{ref} values were equal to η_{water} = 1.33.

Reference:

[1] Zheng, H., Wang, Q., Long, Y., Zhang, H., Huang, X., Zhu, R.,2011. Chem. Commun. 47, 10650–10652.

[2] Eaton, D. F. 1988. Pure & Appl. Chem. 60, 1107–1114.

Fig. S-4

The fluorescence spectra of the C-dots collected after different dialysis times. I_{430} and I_{480} were the PL intensities at 430 nm and 480 nm with excitation at 330 nm and 390 nm, respectively.

After the first two hours: I_{430}/I_{480} = 2.38

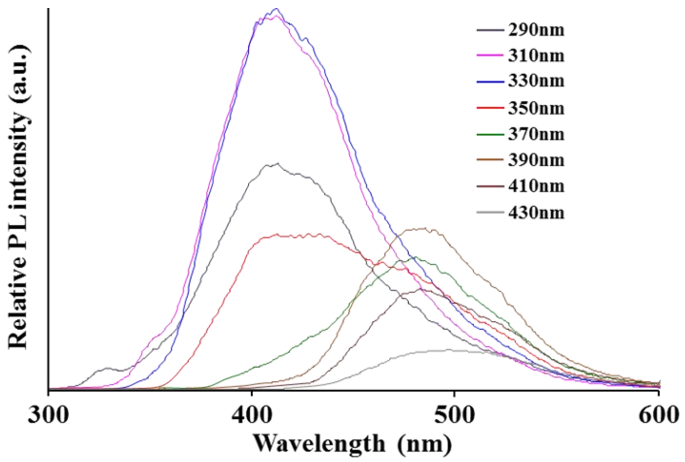

After four hours: I_{430}/I_{480} = 2.17

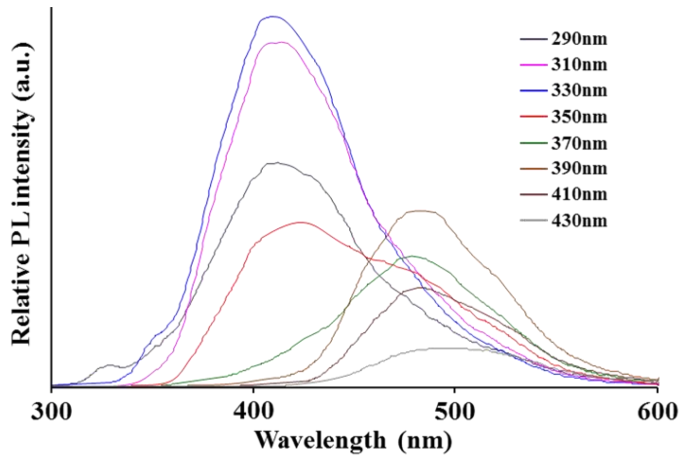

After six hours: I_{430}/I_{480} = 1.74

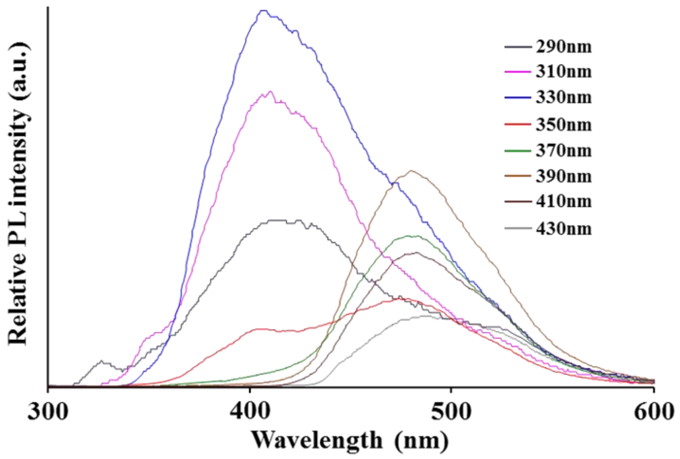

After eight hours: I_{430}/I_{480} = 1.30

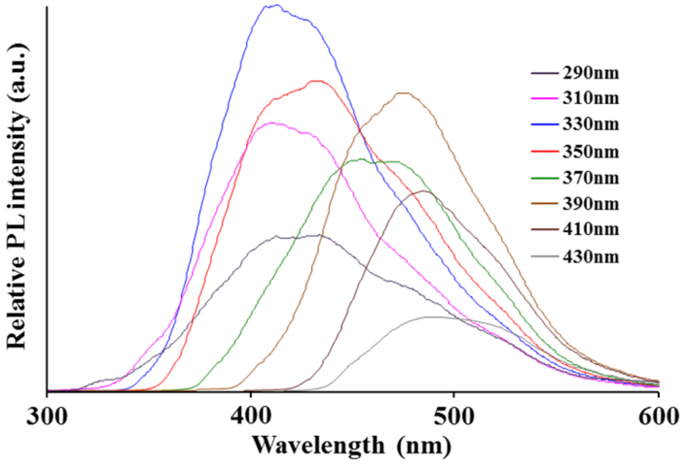

After one day: I_{430}/I_{480} = 1.27

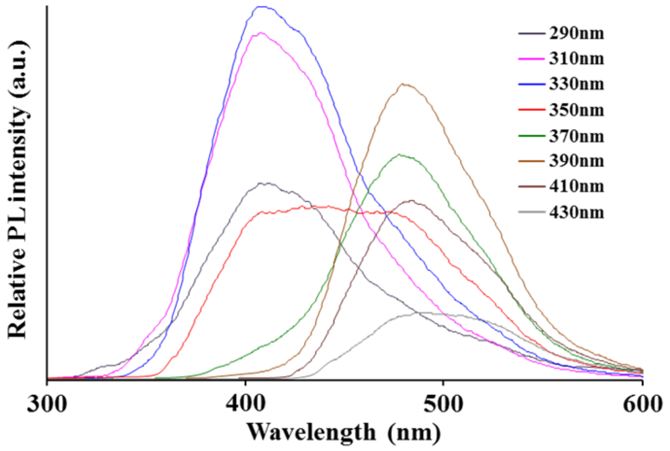

After three days: I_{430}/I_{480} = 0.74

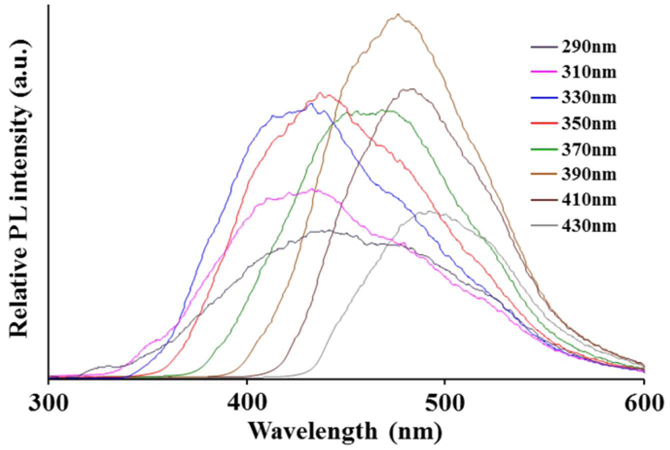

After five days: I_{430}/I_{480} = 0.73

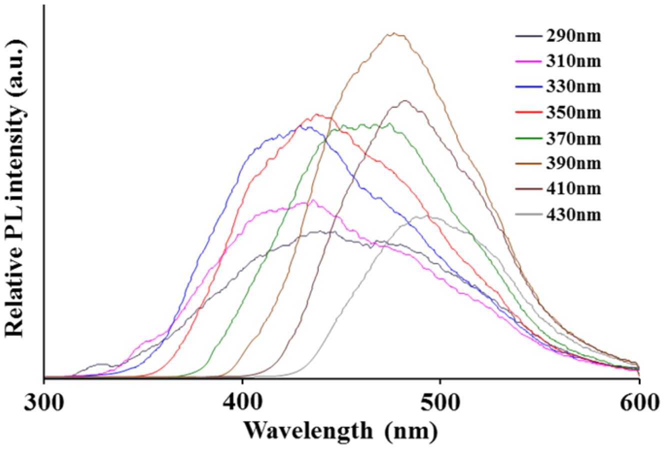

After seven days: I_{430}/I_{480} = 0.69

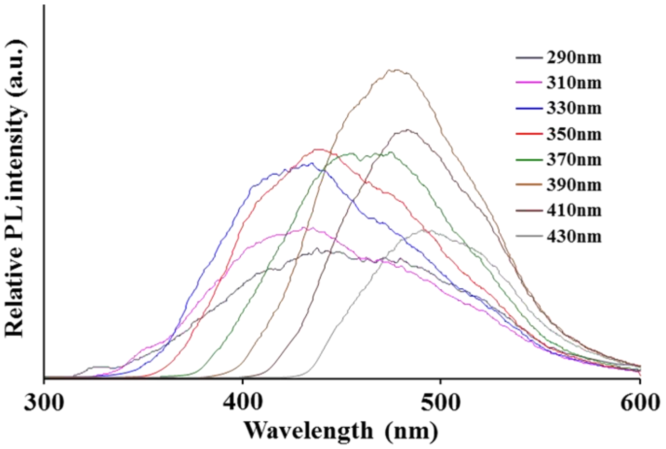

Fig. S-5 TEM images of the C-dots dialyzed for two hours and their size distribution.

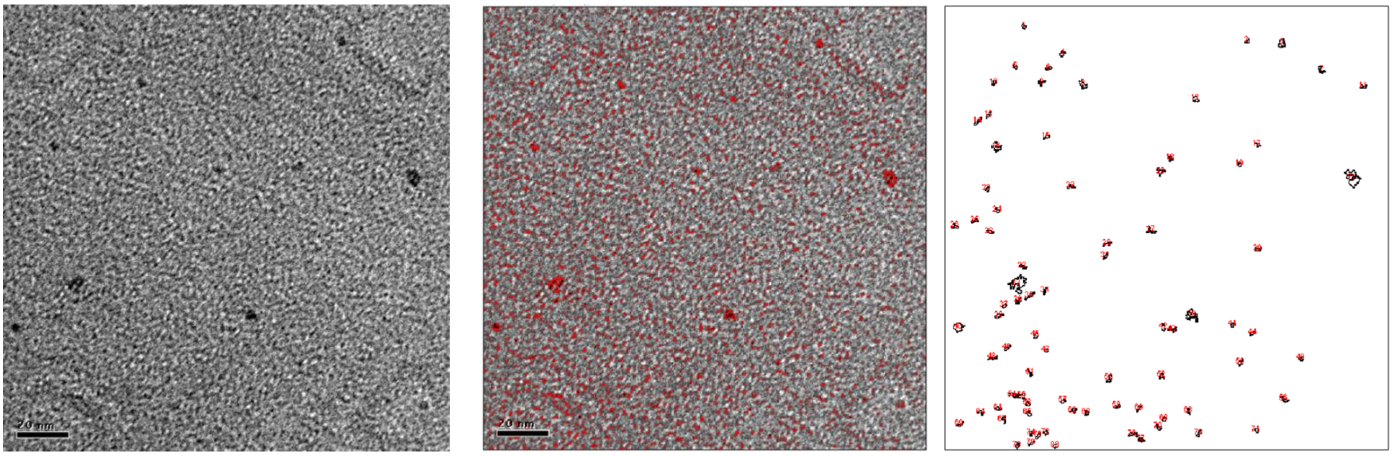

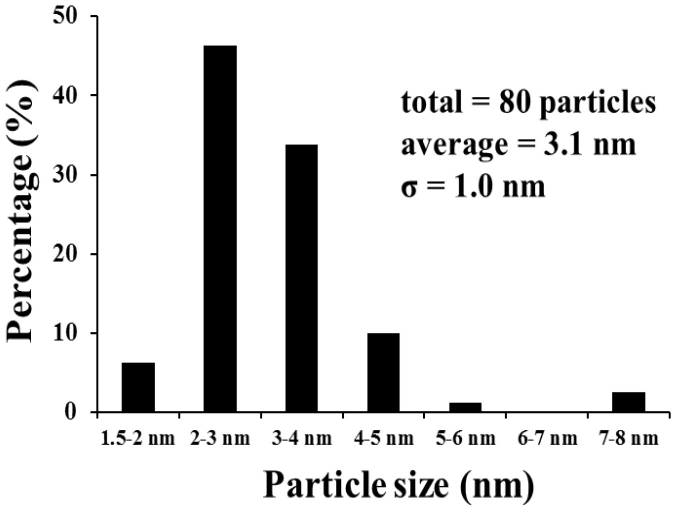

TEM images of the C-dots dialyzed for eight hours and their size distribution.

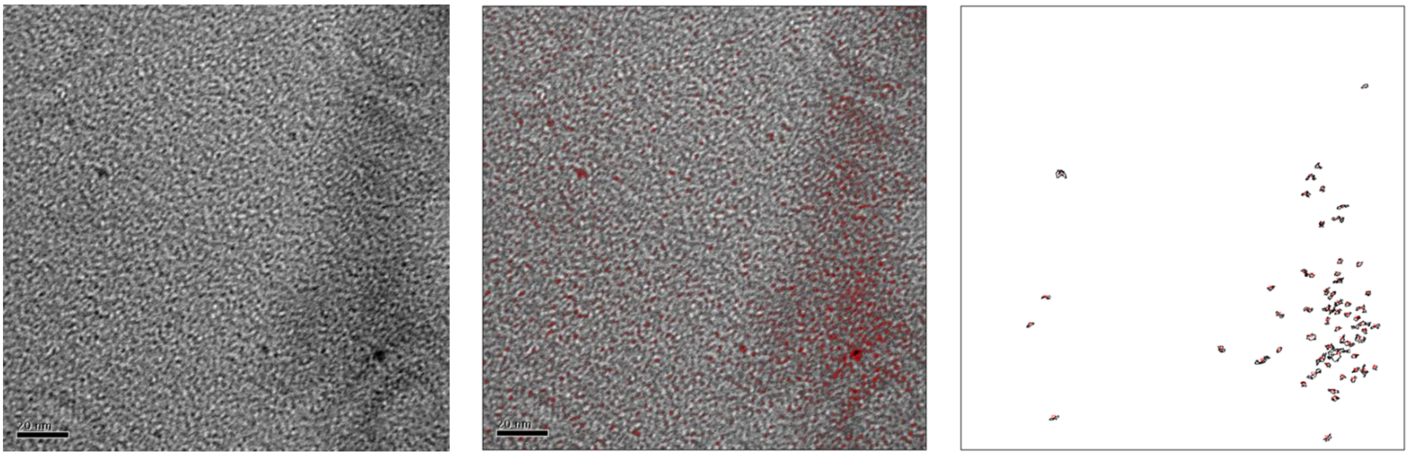

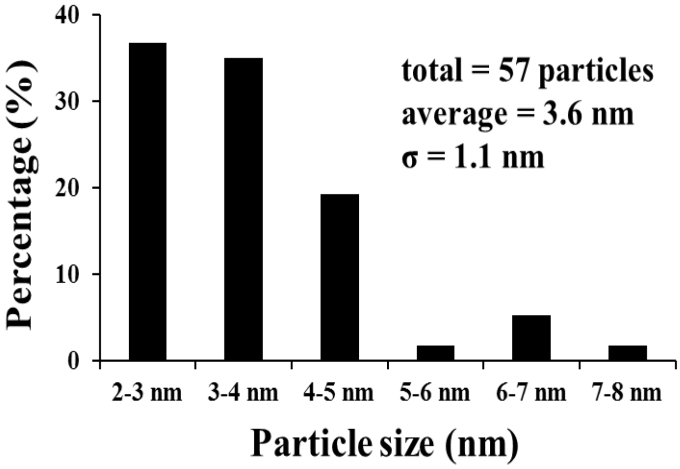

Fig. S-6 Up-conversion emissions of the prepared C-dots under different wavelength excitations.

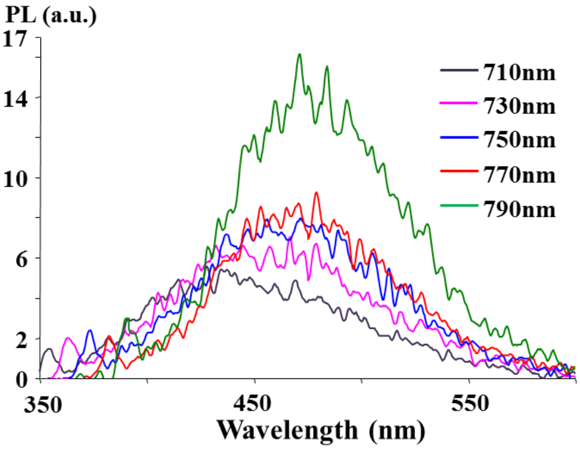

Supplement: Supplementary file 1 [file nanomaterials-08-00372-s001.pdf]
